# Nanoceria as an Efficient and Cost-Effective Metal-Free Catalyst for the Oxidation of Alcohols

**DOI:** 10.1021/acssuschemeng.5c05061

**Published:** 2025-08-15

**Authors:** Claire Squarzoni, Nicolas Kania, Malcolm Dearg, Max Quayle, Hang Hu, Thomas J. A. Slater, Andrea Folli, Alberto Roldan, Marc Pera-Titus, Anne Ponchel

**Affiliations:** † 375020 Univ. Artois, CNRS, Centrale Lille, Univ. Lille, UMR 8181, Unité de Catalyse et de Chimie du Solide (UCCS), Lens F-62300, France; ‡ Eco-Efficient Products and Processes Laboratory (E2P2L), UMR 3464 CNRS-Solvay, Shanghai 201108, P. R. China; § Cardiff Catalysis Institute, School of Chemistry, 2112 Cardiff University, Main Building, Park Place, Cardiff CF10 3AT, U.K.

**Keywords:** cerium oxide, gold, benzyl alcohol, metal-free oxidation, *t*-butyl hydroperoxide

## Abstract

This study investigates the catalytic properties of nanoceria for the liquid-phase oxidation of aromatic and aliphatic alcohols using *t*-butyl hydroperoxide as an oxidant without the need for base or supported noble metals. The morphology, reducibility, and reversible H_2_ adsorption characteristics of ceria were comprehensively studied using X-ray diffraction, BET, HAADF-STEM, H_2_-TPR, H_2_-TPD, and X-ray photoelectron spectroscopy. Radical formation was interrogated by electron paramagnetic resonance (EPR) using dimethyl pyrrolidine *N*-oxide (DMPO) and *N*-*tert*-butyl-α-phenylnitrone (PBN) as spin traps, complemented by atomistic simulations to elucidate the influence of trap and radical adduct adsorption on the catalysts on radical abundance. The solvent played a critical role in enhancing the catalytic performance and carbon balance. The catalyst retained its structural integrity during the reaction in acetonitrile and could be reused for at least five consecutive runs. EPR analysis revealed that peroxyl radicals (*t*Bu-OO^•^) were the predominant reactive species with no detectable formation of oxyl (*t*Bu-O^•^) radicals, ruling out a Fenton-like catalytic mechanism in solution. Incorporating small amounts of Au (0.5–1.0 wt %) as Au­(I) single atoms or clusters reduced the catalytic activity due to a decreased surface reducibility and reversible H_2_ adsorption despite an increased peroxyl radical formation. However, Au doping did not alter the product distribution. Compared to a benchmark 0.3 wt % Au/TS-1 catalyst, nanoceria achieved a 60% cost reduction and an *E*-factor of 0.08 (vs 0.2–1.3 for 0.3 wt % Au/TS-1) at equivalent acid production rates, highlighting the economic and environmental benefits.

## Graphical abstract

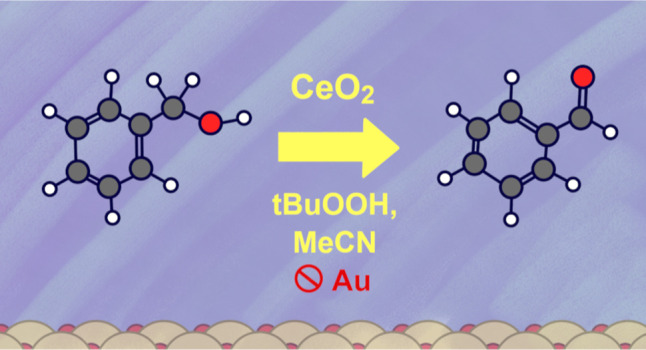

## 1 Introduction

The oxidation of alcohols to carbonyl compounds is a pivotal transformation in biorefineries, enabling the conversion of alcohol and polyol feedstocks into bulk and fine chemicals. Supported noble metal catalysts (e.g., Au and AuPd) have been widely employed for this purpose, utilizing air/O_2_ or H_2_O_2_ as cost-effective and environmentally benign oxidants.^1–5^ The reactions typically proceed in an alkaline aqueous media (pH 9–11), producing water as the primary byproduct. However, postreaction neutralization is required, leading to elevated *E*-factors (mass of waste per unit mass of product) ranging from 0.2 to 1.3 (pH 12–14, H_2_O_2_ as an oxidant).^6^

As alternatives, first-row transition-metal oxides (e.g., MnO_*x*_ and VO_*x*_ FeO_*x*_) and mixed-metal oxides based on Cr, Mn, Co, Fe, or Ni (e.g., spinels and perovskites) have been extensively explored for liquid-phase alcohol oxidation primarily due to their significantly lower cost compared to noble metals [e.g., $90,000 per kg for Au (Q1 2025) vs $3.7 per kg for MnO_2_ (2022 average)].^7,8^ The catalytic properties of metal oxides stem from their redox properties following a Mars-van-Krevelen mechanism.^9–11^ The catalytic performance can be tuned via crystal phase engineering, morphology control, defect chemistry modulation, doping with alkaline/transition metals (e.g., Ni and Co), or by supporting metal oxides on acid–base and redox metal oxides, including zeolites/zeotypes.^12–14^ Despite these advantages, metal oxides frequently suffer from leaching in polar solvents, hindering industrial adoption.

Among rare-earth oxides, nanoceria (CeO_2_, 6–12 nm particles) exhibits intrinsic activity for partial oxidation reactions. While widely recognized as a promoter for supported metal catalysts,^15–19^ nanoceria has been underexplored as a standalone catalyst for liquid-phase oxidations despite its competitive cost [e.g., $1.37 per kg for CeO_2_ (Q2 2025) vs $3.7 per kg for MnO_2_],^8,20^ and ease of recycling via reductive dissolution.^21–24^ Its catalytic properties arise from the labile Ce^3+^/Ce^4+^ redox couple, which facilitates high lattice oxygen mobility, oxygen vacancy formation below 400 °C, and tunable basicity.^16,25–27^ Exposed (110) and (100) facets enhance oxidation activity, as oxygen vacancies on these surfaces activate O_2_ to generate radical species (e.g., superoxide and organic radicals).^5,28,29^ For example, Zr-doped ceria catalyzed the vapor-phase oxidation of benzyl alcohol (BnOH) to benzaldehyde (BnAH) at 350 °C under a 4 mL­(STP)·h^–1^ airflow with 80% BnOH conversion, 82% BnAH selectivity, and high reusability.^30^ Zr- and Mn-substituted ceria catalyzed the aerobic oxidation of vanillyl alcohol (VA) into vanillin (VAL) at 140 °C and 20 bar O_2_ for 4–5 h, resulting in more than 90% VA conversion and 100% VAL selectivity.^31,32^ Nanoceria also enhanced liquid-phase cyclohexanone oxidation to adipic acid using glacial acetic acid as solvent at 118 °C and 15 bar O_2_ with 66% selectivity at 64% conversion.^33^ Self-assembled ceria nanocubes (44–46 nm) based on 6 nm ceria nanocrystals, exposing preferentially (100) planes, achieved complete *p*-xylene conversion into terephthalic acid at 85 °C and 1 bar O_2_, albeit with longer reaction times (>18 h) and ceria loadings (>10% mol).^34,35^

Ceria can also catalyze oxidation reactions with peroxides (e.g., *t*-butyl hydroperoxide or TBHP). However, prior systems exhibited limitations. Ceria microspheres (20–30 m^2^·g^–1^) gave only 18% BnOH conversion and 80% BnAH selectivity.^36^ BnOH was oxidized by ceria nanoparticles supported over mesoporous silica (MCM-41), showing 64% BnOH conversion and moderate selectivity to BnAH (41%) at 90 °C, 1:1 BnOH/TBHP molar ratio and using 10 wt % catalyst.^37^ Nanostructured ceria also oxidized cyclohexane to KA oil (cyclohexanol and cyclohexanone) using TBHP as an oxidant.^38^ Nanocubes outperformed nanorods, achieving full TBHP conversion at a 31% cyclohexane conversion. Finally, sub-15 nm ceria nanowires oxidized aniline to nitrosobenzene (up to 98% selectivity) and 58% conversion using H_2_O_2_ at room temperature.^39^

This work systematically evaluates nanostructured ceria composed of nano-octahedra and truncated polyhedra enriched with {111} facets for base-free, noble-metal-free liquid-phase alcohol oxidation using TBHP. We correlate catalyst properties (morphology, reducibility, and H_2_ adsorption)—probed via X-ray diffraction (XRD), BET, HAADF-STEM, H_2_-TPR/TPD, and X-ray photoelectron spectroscopy (XPS)—with activity, leveraging electron paramagnetic resonance (EPR) spin trapping (DMPO/PBN), and atomistic modeling to elucidate radical pathways. The impact of Au doping (0.5–1.0 wt %) on activity and selectivity is also assessed, alongside a comparative analysis of cost and sustainability metrics against benchmark Au-based catalysts.

## 2 Experimental Section

### 2.1 Materials and Chemicals

Ceria (HSA5, Solvay, 219 m^2^/g, nano-octahedra and truncated polyhedral assembled in agglomerates, Figure S1), titania (TiO_2_, anatase, Sigma-Aldrich, 10 m^2^/g), silica (SiO_2_, Sipernat, 90 m^2^/g), alumina (Al_2_O_3_, Puralox Scca-5/170, Sasol, 154 m^2^/g), and activated carbon (Nuchar SA-20, Ingevity, 1500 m^2^/g) were used as catalysts and catalytic supports. Ammonium carbonate [(NH_4_)_2_CO_3_, 99.9%, Acros Organics] and tetrachloroauric acid (HAuCl_4_, 99.9985% Au, Strem Chemicals) were used for Au@ceria catalyst synthesis. Benzyl alcohol (BnOH, 99%, Fisher Scientific), *tert*-butyl hydroperoxide (TBHP, 70% in water, Sigma), cinnamyl alcohol (CnOH, 99%, Acros Organics), 2-octanol (99.5%, Aldrich), cyclohexanol (99%, Alfa Aesar), 1-phenylethanol (99%, Aldrich), 4-methylbenzyl alcohol (98%, Aldrich), and furfuryl alcohol (99%, Aldrich) were used as reagents for the catalytic tests. Cyclohexanone (99%, Sigma) and 2-octanone (99%, Acros Organics) were used as internal standards for gas chromatography analysis. Acetonitrile (99.5%, Fisher Scientific), *p*-xylene (99%, Acros Organics), toluene (99.7%, Honeywell), dioxane (99.5%, Fisher Scientific), cyclohexane (99.7%, Honeywell), and ethanol (99%, Fisher Scientific) were used as solvents for the catalytic tests.

### 2.2 Preparation of Au@ceria Catalysts

A series of Au@ceria catalysts (0.25–5 wt % Au) were prepared by the deposition–precipitation method.^40,41^ Briefly, 1 g of CeO_2_, either uncalcined or calcined at 400 °C for 4 h in a tubular furnace under a 50 mL­(STP)/min airflow, was homogeneously suspended in 50 mL of deionized water for 1 h under ultrasonic irradiation (Fisher Scientific CL-18, 120 W, 20 kHz, amplitude 60%, pulse 1:1). Then, 25 mL of an aqueous solution of ammonium carbonate (1.5 M) was added, and the suspension was homogenized for another 30 min at room temperature in an ultrasonic bath, followed by vigorous stirring for 20 min. After this period, 25 mL of an aqueous solution of HAuCl_4_ (2 mM for 1 wt % Au) was added dropwise to the suspension, followed by vigorous stirring for 90 min. The suspension was then filtered, and the solid was washed twice with water at 70 °C, dried at 60 °C for 12 h, and calcined at 400 °C for 4 h in a tubular oven under 50 mL­(STP)·min^–1^ airflow. The catalyst samples were labeled as *x* % Au@ceria, where *x* indicates the nominal weight ratio of Au in the catalysts.

### 2.3 Catalyst Characterization

#### 2.3.1 Inductively Coupled Plasma Optical Emission Spectrometry

The bulk Au composition of the catalysts was measured by ICP-OES. Prior to analysis, 10 mg of catalyst was dispersed in 3 mL of an aqueous solution of HCl (37%) and 1 mL of an aqueous solution of HNO_3_ (70%) and was heated to 110 °C for 2 h.

#### 2.3.2 X-ray Diffraction

XRD patterns were recorded on a Bruker D8 Advance apparatus with Bragg–Brentano geometry equipped with a Cu anode (λ = 1.5418 Å) and a 1D PSD Lynxeye detector. The scattering intensities were measured in the range 2θ = 20–80° with a 0.02° step and a 2 s/step speed. The diffraction patterns were indexed to the cubic structure of CeO_2_ (JCPDS card no. 34-0394) and the face-centered cubic structure of Au (JCPDS card no. 04-0784). Crystallite sizes (*D*) were calculated from the Scherrer equation *D* = *K* λ β cos θ, where *K* is the Scherrer constant (0.89), λ is the wavelength of the X-ray beam (1.5418 Å), β is the full width at half-maximum (fwhm) of the peak, and θ is the Bragg angle.

#### 2.3.3 Diffuse Reflectance UV–Vis Spectroscopy

The diffuse reflectance UV–vis spectra of the catalysts were recorded in reflectance mode using a Shimadzu UV-2600 spectrophotometer equipped with a 60 mm integrating sphere. BaSO_4_ was used as a standard. The Schuster–Kubelka–Munk (SKM) absorption function, expressed by *F*(*R*) = (1– *R*_∞_)^2^/2*R*_∞_, was applied for data interpretation, where *R*_∞_ is the reflectance of a thick solid. The UV–vis spectra were acquired between 200 and 800 nm with a 0.5 nm step and a 0.6 s·nm^–1^ speed.

#### 2.3.4 BET Analysis. Porosity Measurements

Nitrogen adsorption–desorption measurements were performed at −196 °C on a Micromeritics TriStar II apparatus. The surface areas were calculated by the Brunauer–Emmett–Teller (BET) method in the relative pressure range 0.05 < *P*/*P*° < 0.35, while the pore size distributions were obtained from the desorption branch using the Barrett–Joyner–Halenda (BJH) method. The total pore volumes were estimated at *P*/*P*° = 0.995, assuming that all the pores were completely filled with liquid nitrogen. Prior to the measurements, the catalysts were outgassed at 100 °C for 12 h under vacuum.

#### 2.3.5 Temperature-Programmed Reduction (H_2_-TPR)

The reducibility of the catalysts was measured by H_2_-TPR on a Micromeritics AutoChem II 2920 system equipped with a thermal conductivity detector to monitor changes in gas composition, a CryoCooler, and a cold trap before the detector for subambient measurements. The H_2_-TPR profiles were measured from −50 to 900 °C, using a heating rate of 10 °C/min under a 5% H_2_/Ar flow (40 mL­(STP)/min).

The reducibility of ceria and Au dispersion in the Au@ceria catalysts was measured using the following expressions:

1 $$reducibility\;\left(\%\right)=\Phi_{H_{2},TPR}^{-50\to 600}\left(\frac{mmol\;H_{2}}{g_{cat}}\right)2\times 10^{-3}\frac{172.11\;\left(g/mol\right)}{\nu_{Ce}}$$

where $\Phi_{H_{2},TPR}^{-50\to 600}$ indicates the amount of H_2_ adsorbed measured by H_2_-TPR from −50 to 600 °C and *ν*_Ce_ designates the stoichiometric number of Ce in CeO_2_.

#### 2.3.6 Temperature-Programmed Desorption (H_2_-TPD)

H_2_ desorption from the catalyst surface was measured on the same apparatus using the following protocol: (1) reduction of the sample at 250 °C under H_2_ flow (40 mL­(STP)/min); (2) cooling to −50 °C under H_2_ flow, equilibration under Ar flow (20 mL­(STP)·min^–1^) at −50 °C for 15 min; and (3) measurement of the TPD profile from −50 to 1000 °C under Ar flow (20 mL­(STP)·min^–1^) using a 10 °C·min^–1^ heating ramp. The percentage of reversible H_2_ (%H_2_ rev) was measured using the following expression:^42^

2 $$\%H_{2}\;rev=\frac{\Phi_{H_{2},TPD}^{bands\;II-VI}}{\Phi_{H_{2},TPR}^{-50\to 600}}\times 100\%$$

The Au dispersion was measured by H_2_-TPD in the range from −50 to 40 °C as follows

$$Au\;dispersion\;\left(\%\right)=\Phi_{H_{2},TPD}^{\mathrm{Band}\;I}\left(\frac{mmol\;H_{2}}{g_{cat}}\right)\frac{2\times 10^{-3}}{\%\;Au\left(g_{Pd}/g_{cat}\right)}196.97{g_{Au}/mol}_{Au}$$ 3

where $\Phi_{H_{2},TPD}^{band\;I}$ and $\Phi_{H_{2},TPD}^{bands\;II-VI}$ refer to the amount of H_2_ desorbed corresponding to band I (<100 °C) and from bands II–VI (100–1000 °C), respectively, characterizing the Au dispersion and the reversible H_2_ storage on ceria, respectively (see more details in the Section 3.4.4, H_2_ reduction).

#### 2.3.7 Thermogravimetric Analysis

Thermogravimetric measurements were performed in a Thermal Analysis System TGA/DSC 3+ from Mettler Toledo equipped with a dynamic gas system. The sample (approximately 20 mg) was placed in an open alumina crucible and heated to 800 °C (5 °C·min^–1^) under an air or nitrogen atmosphere, each with a gas flow of 50 mL·min^–1^.

#### 2.3.8 X-ray Photoelectron Spectroscopy

The surface composition of the different catalysts was analyzed by XPS using a Kratos Axis Ultra DLD apparatus equipped with a hemispherical analyzer and a delay line detector. The spectra were recorded using an Al monochromated X-ray source (10 kV, 15 mA) with a pass energy of 40 eV (0.1 eV·step^–1^) for high resolution spectra and a pass energy of 160 eV (1 eV/step) for the survey spectrum in hybrid mode and slot lens mode, respectively. The Ce 3d binding energy (916.7 eV) was used as an internal reference. Peak fitting and deconvolution of the experimental photopeaks was performed by CasaXPS software. The percentage of surface Au (% Au_surf_) in the Au@ceria catalysts was computed using the expression

4 $$\%\;Au_{surf}=\frac{Au/Ce\left(XPS\right)}{Au/Ce\left(ICP\right)}\times 100\%$$

The Ce 3d XPS spectra were fitted with 10 peaks to estimate the Ce^3+^ percentage on the surfaces of samples. Six photopeaks labeled as (*u*, *v*), (*u*′, *v*′), and (*u*″, *v*″) referring to three doublets of the spin–orbit split components are ascribed to Ce^4+^, while the other four photopeaks labeled as (*v*_0_/*u*_0_) and (*v*′/*u*) are attributed to Ce^3+^. The Ce^3+^ content was calculated using the expression

5 $$\%{\;Ce}^{3+}\;surface=\frac{\left[{Ce}^{3+}\right]}{{[Ce}^{3+}]+{Ce}^{4+}]}\times 100\%$$

where [Ce^3+^] refers to the sum of the areas of *u*′, *u*_0_, *v*′, and *v*_0_ peaks and [Ce^4+^] refers to the sum of the areas of *u*‴ *u*″, *u*, *v*‴, *v*″, and *v* peaks.

#### 2.3.9 Scanning Transmission Electron Microscopy

The Au distribution on ceria was inspected by scanning transmission electron microscopy (STEM). High-angle annular dark-field (HAADF) STEM images were acquired on an aberration (Cs)-corrected JEOL ARM300F microscope by using a 300 kV accelerating voltage. Images were taken on a JEOL HAADF annular detector with 58.3 ± 1 and 216.1 ± 6.4 mrad detector inner-angle and outer-angle, respectively, and 26.2 mrad convergence semiangle. Energy dispersive X-ray spectroscopy (EDXS) spectral maps were acquired on an aberration (Cs)-corrected JEOL ARM200F microscope by using a 200 kV accelerating voltage. EDXS associated HAADF images were taken on a JEOL HAADF annular detector with 34 and 133 mrad detector inner-angle and outer-angle, respectively, and 23 mrad convergence semiangle. EDXS map data was acquired with a JEOL Centurio dual detector, with the sample grids loaded in a low-background beryllium holder. Further EDXS spectrum images were obtained on a Thermo Fisher Scientific Spectra 200 (S)­TEM, using a Thermo Fisher Scientific Super-X detector at a 200 kV accelerating voltage.

### 2.4 Catalytic Tests

The catalytic properties of the nanoceria and Au@ceria catalysts were investigated using the model oxidation reaction between BnOH and TBHP in acetonitrile. In a typical test, the given catalyst (100 mg), BnOH (150 mg, 1.39 mmol), cyclohexanone (200 mg, 2.0 mmol) used as an internal standard, and TBHP (2.07 mmol) were placed in acetonitrile (10 mL) in a 50 mL three-necked flask equipped with a reflux condenser. The reaction was conducted at 60 °C in atmospheric air with magnetic stirring (500 rpm). Sample aliquots were taken from the reaction mixture at different time points, and the unconverted reactant and reaction products were analyzed by gas chromatography using a Shimazu GC2010+ apparatus equipped with a ZB-FFAP 60 m column and an FID detector. The BnOH conversion, selectivity and yield of the products, carbon balance, catalytic activity, and turnover frequency (TOF) were calculated as follows (eqs 6–12)­

6 $$BnOH\;conversion\;\left(\%\right)=\left(1-\frac{n_{BnOH}}{n_{BnOH}^{0}}\right)\times 100$$

7 $${selectivity}_{i}\;\left(\%\right)=\left(\frac{n_{i}}{n_{BnOH}^{0}-n_{BnOH}}\right)\times 100$$

8 $${yield}_{i}\;\left(\%\right)=\left(\frac{n_{i}}{n_{BnOH}^{0}}\right)\times 100$$

9 $$carbon\;loss\;\left(\%\right)=\left(1-\frac{n_{BnOH}}{n_{BnOH}^{0}}-\frac{\sum_{i}n_{i}}{n_{BnOH}^{0}}\right)\times 100$$

10 $$specific\;activity=-\frac{1}{m_{cat}}\cdot {\frac{{dn}_{BnOH}}{dt}|}_{t=0}{[mol\;s}^{-1}{\;g}^{-1}]\left(10\right)$$

11 $$intrinsic\;activity=-\frac{1}{S_{BET}\times m_{cat}}\cdot {\frac{{dn}_{BnOH}}{dt}|}_{t=0}\left[{mol\;s}^{-1}\;{\;m}^{-2}\right]$$

12 $$TOF=2\times \frac{intrinsic\;activity}{\Phi_{H_{2},TPD}^{bands\;II-IV}}\left[s^{-1}\right]$$

where *n* ^0^_BnOH_ and *n*_BnOH_ refer to the initial and final number of moles of BnOH, *n*_*i*_ is the number of moles of the *i*th product, i.e., BnAH and BzOH, *m*_cat_ is the catalyst loading, and *S*_BET_ is the specific surface area of the catalyst.

In some catalytic tests, the amount of residual TBHP after the reaction was measured by iodometry (indirect titration method) as follows: an aliquot of 2 mL taken from the reactor at the end of the test was placed in an Erlenmeyer flask with 10 mL of acetonitrile, 15 mL of a 1 M KI solution, 5 mL of a 1 M H_2_SO_4_, and 5 mL of ammonium heptamolybdate (0.042 M). Then, the liberated iodine was titrated with a 0.1 M standard solution of Na_2_S_2_O_3_. Additional catalytic tests were conducted under similar conditions except that TBHP was replaced by H_2_O_2_ or gaseous dioxygen. In the latter case, pure O_2_ gas was continuously bubbled at a flow rate of 5 mL­(STP)·min^–1^ into the organic solvent using a capillary tube.

Formation of heavy products was investigated using liquid chromatography–mass spectrometry (LC–MS) on a LC-QExactive Mass Spectrometry (Thermo) instrument (Source Type ESI, Scan Begin 50 *m*/*z*, Scan End 1000 *m*/*z*, Ion Polarity Positive).

### 2.5 Catalyst Recycling Experiments

To evaluate the robustness of nanoceria , recycling experiments in the oxidation reaction of BnOH were performed in acetonitrile using TBHP as the oxidizing agent under the typical conditions mentioned above (60 °C and 24 h). After each test, the spent nanoceria was separated from the reaction medium by decantation and washed under ultrasonic treatments in acetonitrile until residual BnOH and reaction products were no longer observed by GC-FID analysis of the resulting supernatant (4 sequential cycles of washing-decantation-supernatant removal). After complete washing, nanoceria was recovered by a final decantation step and dried at 100 °C for 24 h before being reused in the following catalytic run.

### 2.6 Characterization of Radicals

Potential radicals generated during alcohol oxidation were probed by EPR using DMPO and PBN as spin traps. The samples for EPR measurements were prepared by using a consistent procedure. The reaction solutions or suspensions were heated at 60 °C for 2 min, after which either DMPO or a PBN spin trap was added and allowed to react for an additional 2 min. Any ceria catalyst was then filtered from a 50 μL sample before the EPR spectrum. Band continuous wave (CW) EPR spectra were recorded at room temperature, 100 kHz magnetic field modulation frequency, 0.1 mT magnetic field modulation amplitude, 5.12 ms time constant, 10 ms conversion time, and 1.002374 × 10^4^ receiver gain.

### 2.7 Density Functional Theory Calculations

The DFT calculations were carried out using the Vienna Ab Initio Simulation Package (VASP)^43^ at the PBE-D3 level.^44,45^ The plane-wave cutoff energy was set to 500 eV. Slab models for Au(111), representing the Au structures, and the oxygen-terminating CeO_2_(111) surfaces were generated as *p*(4 × 4 × 5) and *p*(4 × 4 × 4) supercells, respectively. Following previous benchmarking work on ceria,^46^ the rotationally invariant DFT + *U* scheme of Liechtenstein et al. was used to help model the electrons in the Ce 4f orbitals,^47^ with a Hubbard correction parameter *U*_eff_ of 4 eV (*U* = 5 eV, *J* = 1 eV). A 20 Å vacuum layer was added to each surface along the *z*-axis. The top two layers of each slab were kept relaxed throughout optimization; the remaining layers were frozen in the optimized bulk lattice. The Brillouin zone was sampled with a 3 × 3 × 1 Monkhorst–Pack mesh.^48^ The implicit solvation model implemented in VASPsol^49^ was used to model bulk effects of acetonitrile (ε_r_ = 37.5).^50^ The binding energy (*E*_B_) of the species is calculated by using eq 13:

13 $$E_{B}=E_{X^{*}}-E_{*}-E_{X}$$

where *E*_X*_ is the ground state energy of the radical trap bound to the surface, *E*_X*_ is the ground state energy of the naked surface, and *E*_X_ is the ground state energy of the free molecule.

## 3 Results and Discussion

### 3.1 Screening of Catalytic Conditions

Initial screening of oxidants at 60 °C for 4 h using 1.39 mmol of BnOH revealed TBHP as the most effective for BnOH oxidation over uncalcined and calcined nanoceria (17% BnAH yield, full carbon balance), outperforming both O_2_ (no conversion) and H_2_O_2_ (2% conversion after 24 h). H_2_O_2_ was unstable under the reaction conditions and rapidly decomposed into H_2_O and O_2_, as evidenced by the gas release immediately after the H_2_O_2_ addition.

Next, we screened various solvents (Figure S2). *P*-xylene, a common solvent for BnOH oxidation, achieved 21% BnOH conversion and 15% BnAH yield after 4 h, albeit with a 3% carbon loss. Toluene affords a slightly lower BnOH conversion (15%) with a higher BnAH yield (19%), likely due to the partial oxidation of toluene itself during the reaction. Cyclohexane shows improved conversion (33%) and a 19% BnAH yield, though it suffers from a high carbon loss (7%). In contrast, dioxane and ethanol exhibits minimal reactivity, with only 7% and 2% BnOH conversion, respectively.

Based on these findings, *p*-xylene and acetonitrile emerge as the most promising solvents. However, further kinetic analysis reveals distinct differences in their long-term performance (Figure 1). In *p*-xylene, BnOH conversion shows a plateau at ca. 80% after 160 h, with only marginal increases beyond this point. In contrast, acetonitrile facilitates rapid conversion, achieving full BnOH conversion within 72 h. Additionally, acetonitrile maintains a 100% carbon balance, whereas *p*-xylene results in 11% carbon loss. Given these advantages, acetonitrile was selected as the optimal solvent for further catalyst screening.

**Figure 1:**
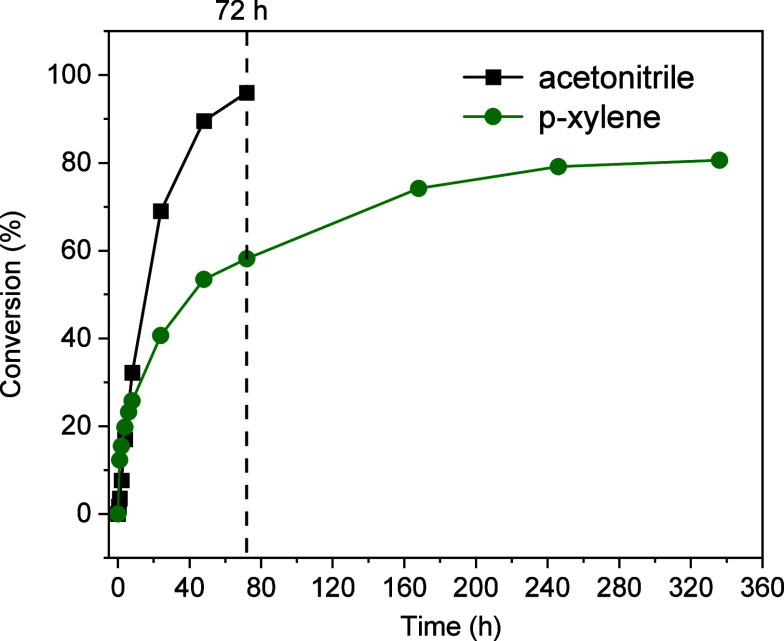
Influence of solvent on BnOH conversion over uncalcined nanoceria. Reaction conditions: 1.39 mmol of BnOH, 100 mg of ceria catalyst, 1.5:1 TBHP/BnOH molar ratio, 60 °C.

Using the optimized solvent (acetonitrile), we evaluated the catalytic performance of various metal oxides, including nanoceria, TiO_2_, SiO_2_, Al_2_O_3_, and activated carbon, at 60 °C for 24 h (Table 1). Uncalcined nanoceria demonstrates superior activity, achieving 69% BnOH conversion with 36% BnAH and 32% benzoic acid (BzOH) yields alongside minimal carbon loss (1%) (Table 1, entry 1). Calcination of ceria at temperatures between 300 and 900 °C progressively reduces the BnOH conversion from 38% to 9% (Table 1, entries 2–5). In comparison, TiO_2_, SiO_2_, and Al_2_O_3_ exhibites significantly lower conversions (10–21%) (Table 1, entries 6–9) with SiO_2_ showing a notable 6% carbon loss. Activated carbon, despite its high surface area (1500 m^2^·g^–1^), delivers only 16% conversion and a 13% BnAH yield (Table 1, entry 5).

**Table 1:** Catalytic Activity of Different Materials (Inorganic Oxides or Others) for BnOH Oxidation^a^

				yield (%)		
entry	catalyst	*S*_BET_ (m^2^·g^–1^)	BnOH conversion (%)	BnAH	BzOH	carbon loss (%)
1	CeO_2_ ^b^	219	69	36	32	1
2	CeO_2_—300 °C^c^	213	38	31	7	<1
3	CeO_2_—500 °C^c^	153	18	17	1	<1
4	CeO_2_—700 °C^c^	55	15	14	1	<1
5	CeO_2_—900 °C^c^	19	6	6	0	<1
6	TiO_2_	10	10	8	0	2
7	SiO_2_	90	21	14	1	6
8	Al_2_O_3_	154	10	8	0	2
9	activated carbon	1500	16	13	2	1

a Reaction conditions: 1.39 mmol of BnOH, 100 mg of uncalcined catalyst, 1.5:1 TBHP/BnOH molar ratio, 60 °C, 24 h.

b Nanoceria (uncalcined).

c Calcination temperature.

These results highlight the exceptional catalytic efficiency of uncalcined nanoceria for BnOH oxidation, outperforming other tested materials in both conversion and selectivity. Given its promising performance, further studies were conducted to explore its applicability in the oxidation of other industrially relevant alcohols.

### 3.2 Alcohol Oxidation on Nanoceria

#### 3.2.1 Kinetics of BnOH Oxidation

The kinetics of BnOH oxidation over nanoceria in acetonitrile at 60 °C was systematically investigated (Figure 2). The reaction initially proceeds with high selectivity toward BnAH, which forms exclusively within the first 7.5 h. Beyond this point, the BnAH yield gradually decreases as it further oxidizes to BzOH, with minimal carbon loss observed throughout the process. After 70 h, the reaction reaches near-complete BnOH conversion, yielding 80% BzOH and 20% BnAH. The apparent activation energy for BnOH oxidation was determined to be 65 kJ·mol^–1^ within the temperature range of 40–80 °C (boiling point of acetonitrile) (Figure S3), a value consistent with literature reports for Au-supported catalysts.^51,52^

**Figure 2:**
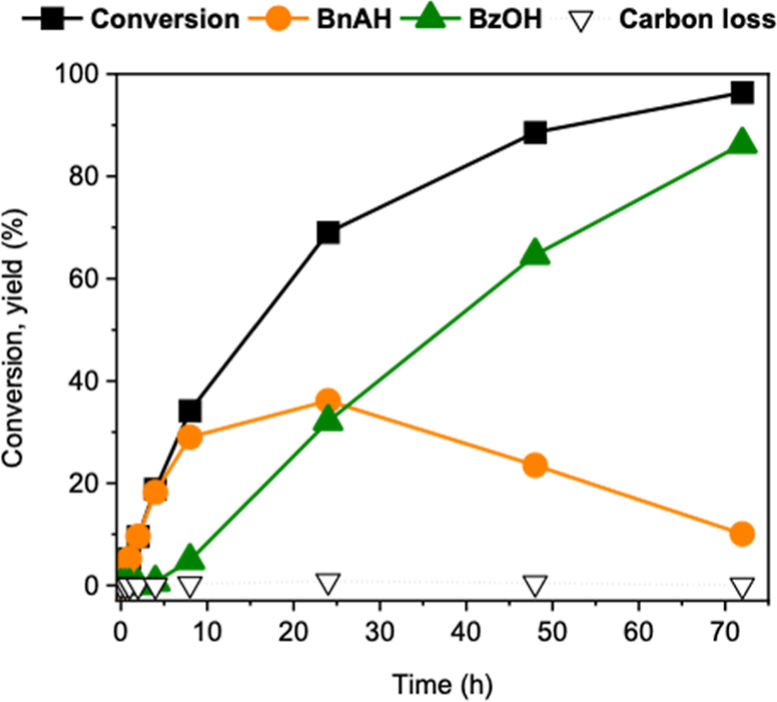
Kinetic profiles of BnOH oxidation to BnAH and BzOH. Reaction conditions: 1.39 mmol of BnOH, 100 mg of nanoceria (uncalcined), 1.5:1 TBHP/BnOH molar ratio, 60 °C.

The intrinsic activity of uncalcined ceria is 1.10 mmol·m^–2^·h^–1^ (or 0.24 mmol·g^–1^·h^–1^ in specific activity), with a TOF of 1.10 h^–1^. The TOF calculation was based on the density of surface ceria sites (0.61 sites·nm^–2^), derived from H_2_-TPD analysis (bands II–IV). This value aligns with the range (0.41–61 sites·nm^–2^) reported by Wachs et al. for metal oxides using methanol chemisorption.^53^

To probe the reaction pathway further, BnAH oxidation was conducted in the absence of BnOH (Figure S4). Unlike the parent reaction, this process exhibits significant carbon loss (21%), suggesting the formation of unidentified heavy byproducts (detected by LC–MS, Figure S5). However, when BnAH oxidation is performed in the presence of an equimolar amount of BnOH and BzOH (Figure 3), the carbon loss decreases to 3–4%, indicating that both BnOH and BzOH inhibit side reactions involving BnAH and TBHP.

**Figure 3:**
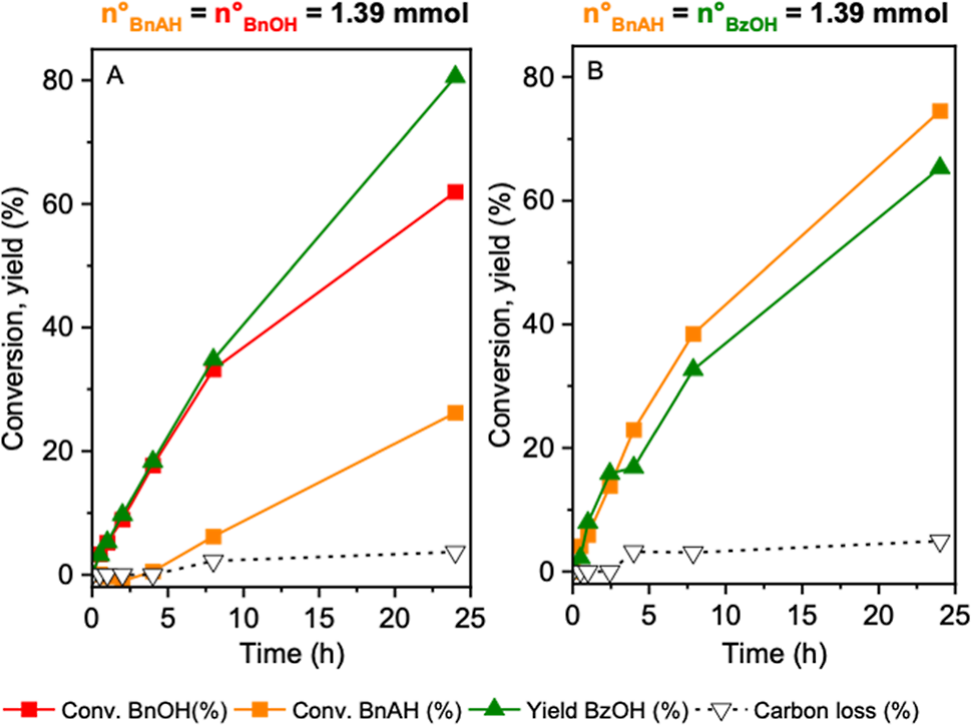
Kinetic profiles of BnAH oxidation with equimolar (A) BnOH and (B) BzOH. Reaction conditions: 1.39 mmol of BnAH, 100 mg of nanoceria (uncalcined), 1.5:1 TBHP/substrate molar ratio, 60 °C.

#### 3.2.2 Recyclability of Ceria

The recyclability of nanoceria was evaluated over five consecutive 24 h runs at 60 °C (Figure 4). After each run, the catalyst was recovered by centrifugation, thoroughly washed to remove organic residues (as verified by GC-FID), and dried at 100 °C for 24 h. While a slight deactivation occurs after the first run, the BnOH conversion remains stable after the third run, maintaining more than 50% BnOH conversion with a 2–4% carbon loss. The particles after the fifth cycle were recovered by centrifugation, allowing us to estimate that about 5% of the catalyst was lost during recycling. This loss of catalyst should contribute to some extent to the slight decrease in the conversion.

**Figure 4:**
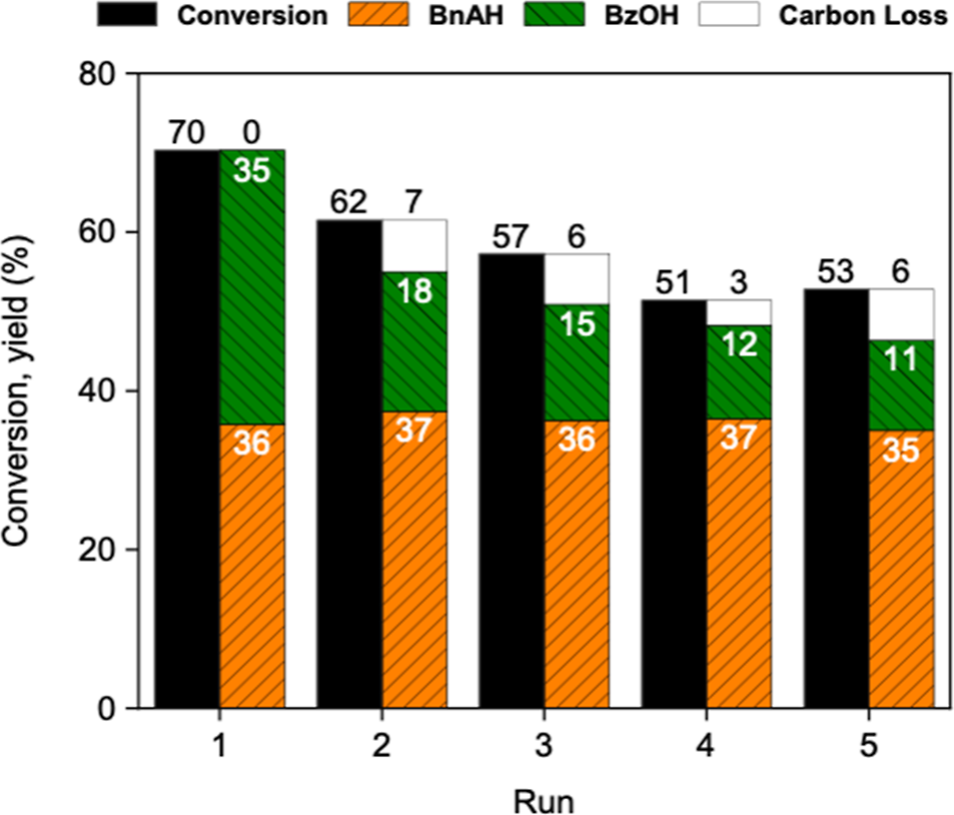
Recyclability of uncalcined nanoceria over five consecutive runs. Reaction conditions: 1.39 mmol of BnOH, 100 mg of catalyst, 1.5:1 TBHP/BnOH molar ratio, 60 °C, 24 h.

Thermogravimetric analysis (TGA) of the spent catalyst after the fifth run (Figure S6) reveals irreversible adsorption of heavy organic species, evidenced by weight loss between 165 and 270 °C. These residues persist even after washing and were further confirmed by TGA under N_2_ (Figure S7). The spent catalyst also shows a modest reduction in surface area (219 → 174 m^2^·g^–1^) and pore volume (0.140 → 0.122 cm^3^·g^–1^), though the pore size remains unchanged at 2.9 nm (Figure S8). The residues can be removed after air-calcination at 400 °C, allowing full regeneration of nanoceria. This is evidenced by the measurement of the specific surface area of the recalcined used ceria, which shows that the value of the initial nanoceria is practically recovered after being subjected to temperature (243 m^2^/g). Further analysis of carbon deposits and a potential change of the surface reducibility of nanoceria during the reaction and upon regeneration is provided in Section 3.4.6.

#### 3.2.3 Economic and Environmental Credentials of Nanoceria

Despite a lower TOF (1.10 h^–1^) compared to a benchmark 0.3 wt % Au/TS-1 catalyst (∼175 h^–1^),^52^ nanoceria’s significantly lower cost ($1.37 per kg vs $272 per kg) translates to ∼60% savings for equivalent BzOH production. For instance, producing 1 ton day^–1^ of BzOH would cost $2283 with ceria vs $3702 with Au/TS-1. Additionally, the *E*-factor for nanoceria-catalyzed reactions (0.08) is markedly lower than that of Au/TS-1 (0.2–1.3), underscoring its environmental superiority, especially under base-free conditions.

#### 3.2.4 Scope of Alcohol Substrates

Nanoceria was further tested for the oxidation of a scope of aromatic and aliphatic alcohols at 60 °C for 24 h using TBHP as an oxidant (Table S1). Nanoceria is active in oxidizing 4-methylbenzyl alcohol (Table S1, entry 2) with 29% and 25% yield of the aldehyde and acid, respectively, and 11% carbon loss. Ceria also oxidizes phenylethanol resulting in 58% yield of the ketone product at 100% selectivity and negligible carbon loss (Table S1, entry 3). For cinnamyl alcohol, cinnamaldehyde is the main product, with a 28% yield at 67% conversion (Table S1, entry 4). BnAH and BzOH are also generated with 20% carbon loss. The auto-oxidation of the as-generated BnAH can explain this observation in the presence of cinnamyl alcohol. To discourage this side reaction, a catalytic test was carried out for cinnamyl alcohol oxidation in the presence of BnOH (Table S1, entry 5, and Figure S9). The presence of BnOH reduces the carbon loss from 20% to 14%, suggesting partial stabilization of cinnamyl alcohol and cinnamaldehyde against parasite degradation and further radical oxidation. Nanoceria is also active for the oxidation of unactivated alcohols, such as cyclohexanol and 2-octanol being selected as model alcohol molecules from plant biomass sources, resulting in the formation of cyclohexanone and 2-octanone with 35% and 26% yields, respectively, and 100% selectivity (Table S1, entries 6 and 7).

### 3.3 Effect of Au Doping on the Catalytic Properties of Ceria

The results above point out that metal-free nanoceria effectively catalyzes BnOH oxidation with TBHP, reaching 69% and 52% before and after calcination, respectively, after 24 h reaction. However, low Au loadings (0.25–0.50 wt %) significantly suppress activity (30–33% conversion) (Figure 5A and Table S2, entries 1–7), suggesting Au disrupts the Ce^3+^/Ce^4+^ redox cycle. At higher Au loadings (1–5 wt %), conversion improves (37–45%) but remains below the value measured on pure nanoceria, indicating only partial compensation. It is worth noting that the drop in activity of Au@ceria catalysts, particularly when small amounts of Au were added (below 1 wt %), cannot be related to metal leaching from ceria. Indeed, comparison between the fresh and spent 1% Au@ceria catalyst did not show any variation in the Au metal loading during the reaction, as evidenced by ICP analysis (Table S2, entries 5 and 8).

**Figure 5:**
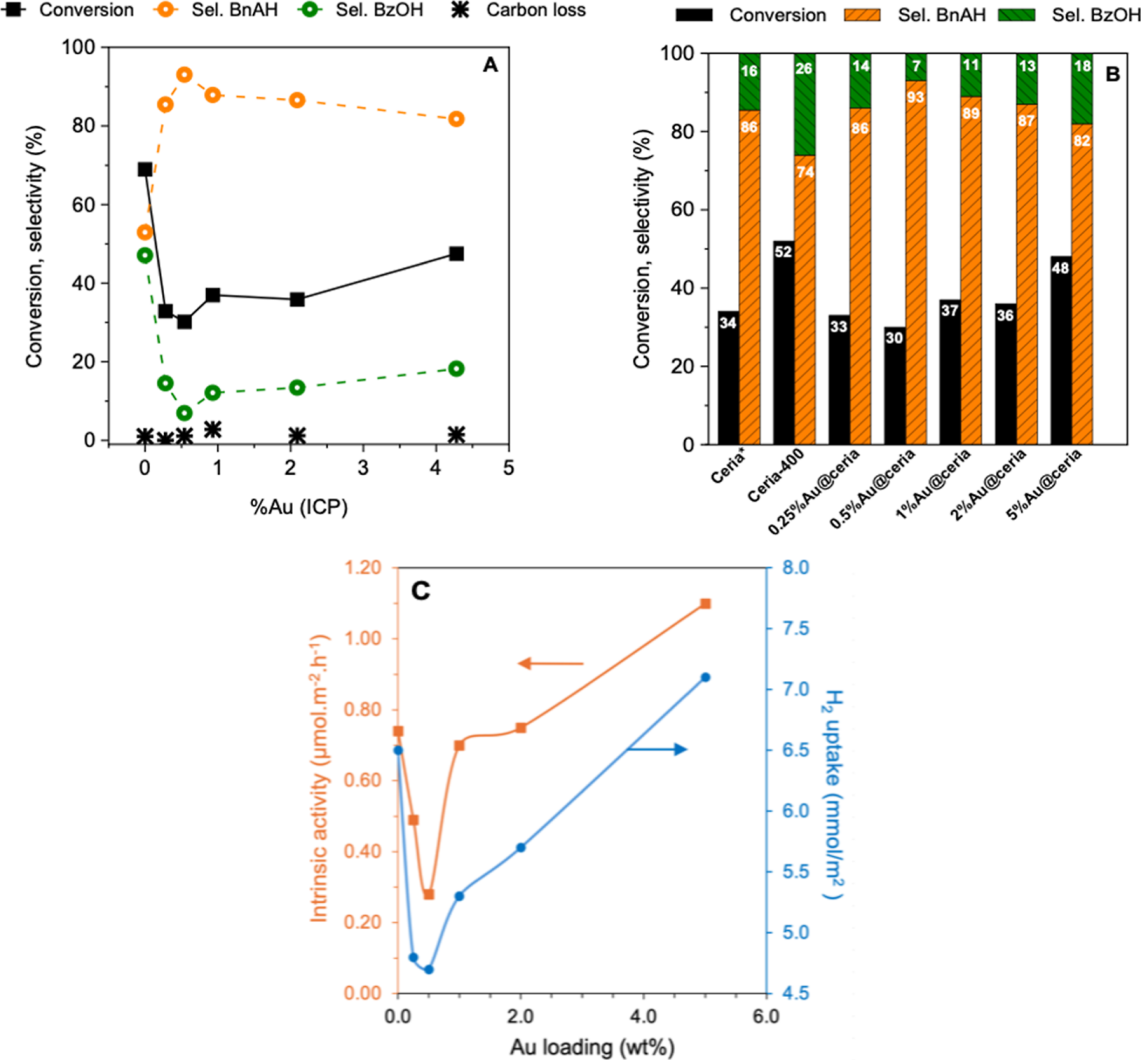
(A) Catalytic performance of nanoceria and Au@ceria catalysts of variable Au loadings in the oxidation of BnOH to BnAH and BzOH; (B) product selectivity results determined at near iso-conversion (30–52%); and (C) correlation between the catalytic activity and H_2_ uptake of Au@ceria catalysts. Reaction conditions: 1.39 mmol of BnOH, 100 mg of catalyst, 1.5:1 TBHP/BnOH molar ratio, 60 °C, 24 h (except 8 h for ceria* in B).

For all catalysts, BnAH is the most abundant product with a very low carbon loss (<3%) (Figure 5A). The BnAH selectivity is 53% for uncalcined ceria and increases to 74% after calcination at 400 °C. Such an increase is explained by the lower BnOH conversion after calcination (52% vs 69%). Calcination at 400 °C exerts little effect on the catalytic activity, exhibiting rather good overlap with the selectivity-conversion curve (Figure S10). Adding Au to ceria results in a higher BnAH selectivity than the parent uncalcined ceria (range of 82–93%), which remains almost unchanged with the Au loading. This observation can also be explained by the lower BnOH conversion after Au doping. Selectivity plots for the different catalysts at BnOH isoconversion (30–52%) reveal no apparent change of selectivity (Figure 5B), suggesting the same catalytic mechanism regardless of the Au loading. Overall, this body of results points out that metal-free nanoceria is active for BnOH oxidation and that Au doping partially inhibits the catalytic activity.

For comparison, we examined Au doping effects on TiO_2_, revealing a fundamentally different behavior from that of nanoceria. Undoped TiO_2_ exhibits minimal activity (10% BnOH conversion) (Table 1, entry 2), while 2 wt % Au doping enhances the BnOH conversion to 29% (consistent with literature^54^) (Table 1, entry 8). This positive contrast with nanoceria—where 2 wt % Au decreases conversion from 52% (calcined CeO_2_) to 37% (2% Au@CeO_2_)—highlights the crucial role of the Ce^3+^/Ce^4+^ redox cycle in ceria’s catalytic mechanism. The opposing trends confirm that low Au loadings specifically inhibit ceria’s redox activity while promoting TiO_2_’s performance. Notably, both systems achieve similar BnAH selectivities (∼88–89%), suggesting parallel reaction pathways despite their divergent responses to Au doping.

### 3.4 Characterization of Au@ceria Catalysts

Given the catalytic results on nanoceria, we conducted a complete characterization study of the parent nanoceria and Au@ceria catalysts by combining XRD, DRUV–vis, HR-TEM, and XPS to rationalize the Au speciation and the effect of Au species on the Ce^3+^/Ce^4+^ pump and catalytic mechanism.

#### 3.4.1 Specific Surface Area

The porous nature of nanoceria with/without Au was studied by N_2_ physisorption at −196 °C. The BET specific surface areas are listed in Table S3. All samples display Type IV isotherms with H2-type hysteresis (Figure S11A), characteristic of a mesoporous network consisting of interparticle pores. The samples exhibit narrow pore size distributions centered at 2.9–3.0 nm (Figure S11B). The specific surface area of pure nanoceria is 219 m^2^/g (Table S3, entry 1), showing a minimal change upon calcination at 400 °C (208 m^2^·g^–1^) (Table S3, entry 2). Deposition–precipitation of Au (i.e., before ceria calcination) barely affects nanoceria’s textural properties (213 m^2^·g^–1^) (Table S3, entry 7), pointing out no pore blockage by Au, even at elevated metal loadings.

#### 3.4.2 XRD Patterns

Figure 6A shows the XRD patterns for the different Au@ceria catalysts. All catalysts exhibit characteristic CeO_2_ fluorite reflections at 2θ angles of 28.5° (111), 33.1° (200), 47.5° (220), 56.3° (311), 59.1° (222), and 69.4° (400). Table S3 lists the ceria (111) crystallite sizes (nano-octahedra) estimated from the Scherrer equation at 2θ = 28.5°. The ceria particle size is about 4.3 nm and remains unchanged after Au deposition, which is consistent with the similar specific surface area of the different samples (Table S3), confirming structural stability upon Au incorporation. No reflections belonging to Au are observed in the range of 0.25–2 wt % Au, pointing out highly dispersed Au species over ceria. In contrast, for 5% Au@ceria, a small reflection appears at 2θ = 37.5° that can be attributed to the (111) planes of Au(0) (Figure 6B).

**Figure 6:**
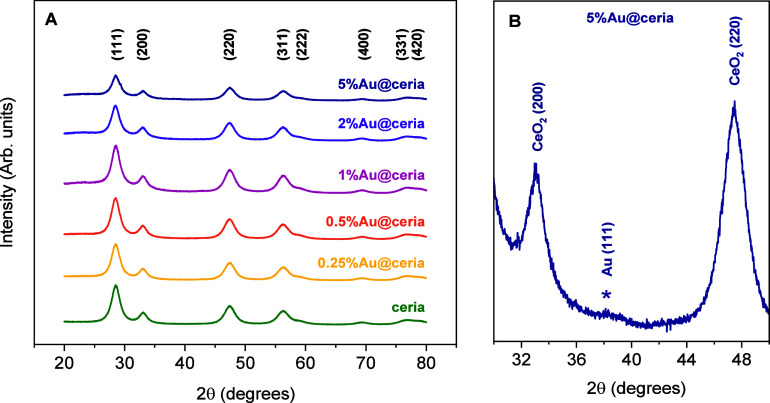
(A) XRD patterns of ceria and Au@ceria and (B) magnified XRD pattern in the 2θ range 30°–50° for the 5% Au@ceria material showing a small peak associated with Au.

#### 3.4.3 DRUV–Vis

DRUV–Vis spectroscopy reveals the electronic structure evolution of Au@ceria catalysts (Figure S12). The DRUV–vis spectrum of pure ceria (profile a) displays two main contributions below 400 nm: (1) a dominant O^2–^ → Ce^4+^ charge transfer band centered at 275 nm and (2) a band near 360 nm that can be assigned to interband transitions.^55,56^ A shoulder is observed at ca. 240 nm that can be attributed to O^2–^ → Ce^3+^ charge transfer, with an adsorption edge at 400–450 nm. These ceria-specific transitions remain essentially unchanged across all Au loadings (0.25–5 wt %, profiles b to f). The emergence of a broad surface plasmon resonance (SPR) band at 575 nm exclusively in the 5 wt % Au sample (see star on profile f),^57^ accompanied by a visible color change to dark black, confirms the formation of metallic Au(0) nanoparticles at higher loadings that is consistent with XRD observations (Figure 6B). In contrast, samples with ≤2 wt % Au loading maintained an absorption edge below 450 nm and showed no SPR feature, suggesting the predominance of oxidized Au species. The progressive color change from yellow (pristine ceria) to gray (low Au loading) to black (5 wt % Au) provides visual confirmation of these electronic structure modifications.

#### 3.4.4 H_2_-Reduction

The H_2_-TPR profile of pristine ceria, whether calcined or not, displays two bands centered at around 500 and 850 °C (not shown), due to the reduction of surface and bulk ceria, respectively (Figure 7A). The first band can be deconvoluted into two bands centered at 445 and 535 °C attributed to surface Ce­(III)–OH^58,59^ and Ce­(IV)–H^60,61^ species resulting from homolytic and heterolytic H_2_ dissociation pathways, respectively, whereas the second band is ascribed to subsurface hydride diffusion.^62–64^ Matching earlier studies,^65,66^ Au incorporation significantly modifies the reduction behavior, lowering the surface reduction temperature from 180 to 60 °C depending on the Au loading (0.25–5 wt %), demonstrating the role of Au in activating H_2_ dissociation. The reduction profiles evolve from narrow/symmetric (≤1 wt % Au) to broad/asymmetric (≥2 wt % Au), indicating increasing heterogeneity of Au species and reduction pathways.

**Figure 7:**
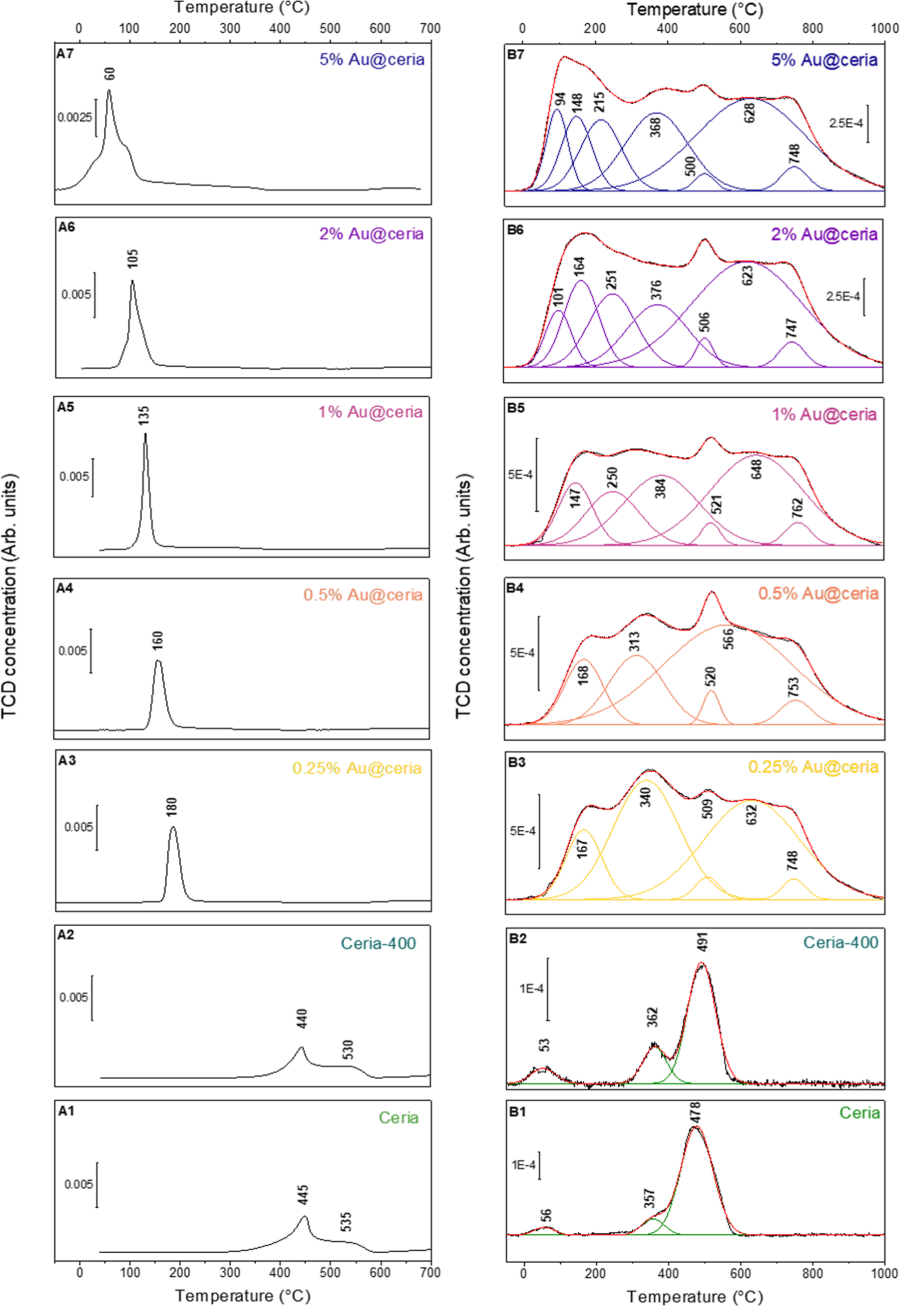
(A) H_2_-TPR profiles and (B) H_2_-TPD profiles of ceria and Au@ceria catalysts: ceria (A(1), B(1)), ceria-400 (A(2), B(2)), 0.25% Au@ceria (A(3), B(3)), 0.5% Au@ceria (A(4), B(4)), 1% Au@ceria (A(5), B(5)), 2% Au@ceria (A(6), B(6)) and 5% Au@ceria (A(7), B(7)).

Quantitative analysis of H_2_-TPR profiles reveals that calcination at 400 °C decreases H_2_ uptake from 1.42 to 1.22 mmol·g^–1^ (6.5 to 5.9 μmol·m^–2^) (Table S4, entries 1–2). From these values, the maximum density of the O vacancies in uncalcined nanoceria is 11 molO % or 3.61 vacancies·nm^–2^ upon reduction that is compatible with ceria particles with preferentially exposed {111} facets. Au doping on ceria-400 initially further reduces uptake (1.10 mmol·g^–1^ for 0.5 wt % Au, Table S4, entries 2–4) before increasing to 1.30 mmol·g^–1^ (7.1 μmol·m^–2^) at 5 wt % Au (Table S4, entries 5–7), resulting in a decrease of O vacancies to 2.92 molO % or 0.90 vacancies·nm^–2^ with a further increase to 4.64 molO % or 1.81 vacancies·nm^–2^. This H_2_ uptake trend correlates directly with catalytic activity in BnOH oxidation (Figure 5C), confirming the essential role of Ce^4+^ and O vacancies in the TBHP-mediated oxidation mechanism.

This observation points out that Ce­(IV) plays a key role in the catalytic oxidation mechanism in the presence of TBHP. The H_2_ uptake of Au@ceria catalysts scales with the BnOH conversion in the oxidation tests as a function of the Au loading­(Figure 5C).

#### 3.4.5 H_2_-Desorption

H_2_-TPD was used to elucidate the interaction between Au species and ceria in the Au@ceria catalysts (Figure 7B and Table S4). TPD measurements were performed by first prereducing the samples at 250 °C under H_2_, cooling to −50 °C under H_2_ flow, and further equilibration under Ar atmosphere at −50 °C, before increasing to 1000 °C. Pristine nanoceria shows a band centered at 478 °C together with a shoulder at 357 °C that can be attributed to reversible H_2_ stored in ceria. The amount of H_2_ released is only 0.11 mmol·g^–1^ (0.51 μmol·m^–2^), accounting for 7% of the H_2_ uptake during reduction (Table S4, entry 1), and corresponding to 1.02 μmol·m^–2^ or 1.6 mol %O or 0.30 Ce­(III)–OH groups·nm^–2^. A smaller band is also observed at lower temperatures (56 °C) with 6 mmol·g^–1^ (0.03 μmol·m^–2^) H_2_ release due to fast H_2_ desorption from the Ce­(III)–OH species on the ceria surface. Further calcination of the sample at 400 °C does not significantly alter the H_2_ desorption pattern with similar desorption releases (Table S4, entry 2).

The H_2_ desorption profile between 25 and 900 °C changes drastically after 0.25–5 wt % Au addition (Table S4, entries 3–7), expanding with multiple desorption bands. For all Au@ceria catalysts, three bands are visible at about 515, 630, and 750 °C with similar intensity. In contrast, the H_2_ desorption profiles between 25 and 400 °C are strongly influenced by the Au loading, encompassing complete suppression of the native band at 56 °C most likely due to Au poisoning of labile Ce­(III)–OH species on the ceria surface,^19^ and the appearance of two new desorption bands at 170 and 330 °C at lower Au loading (i.e., 0.25 and 0.50 wt %). These additional bands shift to lower temperatures at higher Au loadings. In particular, the H_2_-TPD profiles of 2% Au@ceria and 5% Au@ceria exhibit a band at about 90–100 °C that is indicative of H_2_ desorption from Au(0).^67^

For all Au@ceria catalysts, the H_2_ release is about 0.74–0.76 mmol·g^–1^ (3.3–4.1 μmol·m^–2^) encompassing 12–13 mol % of reversible H_2_ storage through Ce­(III)–OH groups or 4.0–5.0 groups ·nm^–2^ (Table S4, entries 3–7). When comparing the H_2_ release (H_2_-TPD) and H_2_ uptake (H_2_-TPR), the reversible H_2_ increases, whereas it decreases at higher loadings until 58% for 5% Au@ceria.

Overall, the band multiplication in the H_2_-TPD profiles after Au doping is indicative of different H_2_ desorption pathways over reduced ceria by recombination/oxidation of Ce­(III)–OH species and diffusion/reduction of subsurface Ce­(IV)–H species in the vicinity of Au species. This comprises potential back H-spillover in the presence of Au clusters/nanoparticles at higher Au loading.

#### 3.4.6 X-ray Photoelectron Spectroscopy

XPS was used to inspect the nature of Au species and the ceria reducibility for Au@ceria catalysts doped with 0.5% Au, 1% Au, and 5% Au. The XPS spectra of the Ce 3d core level for the different samples exhibit six spin–orbit coupling levels corresponding to the 3d_5/2_ (*v*) and 3d_3/2_ (*u*) states corresponding to three pairs of doublets (*u,v*), (*u*′,*v*′), and (*u*″ *v*″) (Figure S13, left). The *v* components show binding energies (BE) at 882.7 eV (*v*), 888.5 eV (*v*″), and 898.3 eV (*v*‴), while the *u* components show BEs at 901.3 eV (*u*), 907.3 eV (*u*″), and 917.0 eV (*u*‴).^68–70^ The Ce^4+^ oxidation state, characterized by a broad *u*‴ band, is predominant for all the samples. The presence of *v*′ and *u*′ components centered at about 883 and 903 eV, respectively, combined with large bands for *v*‴ and *u*‴ components, is indicative of Ce^3+^ on the ceria surface.^71^ The spectra are very similar for all samples and do not display visible *v*′ and *u*′ components after deconvolution. This indicates that Au doping and calcination do not exert any remarkable effect on the distribution of the surface Ce^3+^/Ce^4+^ species. Regardless of the Au loading, the samples show a Ce^3+^ surface concentration of 18–24% (Table S3).

The XPS spectra of the O 1s core level for the parent ceria and the three Au@ceria catalysts can be deconvoluted into 4 bands (Figure S13, right). Bands (A) and (B) centered at 529 and 530 eV are attributed to lattice O in the ceria network and O species bound to Ce^3+^, respectively.^72^ Bands (C) and (D) centered at 532 and 533 eV are attributed to weakly bound O (e.g., surface hydroxyl groups and vacancies) and either carbonate or adsorbed H_2_O.^73^ The intensity of the D signal decreases after Au loading, which can be explained by removing adsorbed carbonate and water species from the ceria surface during calcination.

Additional XPS investigations were conducted on the used ceria, thereby confirming the loss of surface carbon after air-calcination without an apparent change of the surface state of nanoceria (Figure S14). Ceria exists primarily as Ce^4+^ with the coexistence of Ce^3+^. The surface concentrations measured by XPS spectra reveal similar molar ratios for pristine and used nanoceria (2.11–2.32), whereas this ratio is lower for used nanoceria after calcination (1.99) (Table S5), suggesting a higher reduction. The O 1s spectra display different features, which depend on both the lattice oxygen and chemisorbed oxygen species. The binding energy at ca. 533 eV (D signal) is assigned to adsorbed oxygen species from adsorbed organic components (e.g., carbonate, hydroxyl, water, etc.), whereas the binding energy at ca. 529 eV (A signal) is ascribed to lattice oxygen. The molar surface ratio of O_D_ to O_A_ (O_D_/O_A_) was calculated according to the peak deconvolution results shown in Table S5. Surface-adsorbed oxygen species from organic residues are present in higher abundance on the used nanoceria than on pristine nanoceria and used nanoceria after air-calcination. Following calcination in air at 400 °C, the disappearance of contribution D is complete.

The XPS spectra of the Au 4f core level for the three Au@ceria samples show the presence of different Au species, with a surface concentration that strongly depends on the Au loading (Figure S15). For 0.5% Au@ceria, only Au­(I) is observed (Figure S15, spectrum a). In contrast, for 1% Au@ceria, Au­(III) is observed in addition to Au­(I) (Figure S15, spectrum b). Finally, for 5% Au@ceria, Au(0), Au­(I), and Au­(III) are observed with a lower atomic percentage of Au­(I) (59%), while the atomic percentage of Au(0) and Au­(III) is 32.2% and 8.8%, respectively (Figure S15, spectrum c). The presence of different Au species at higher Au content is consistent with the H_2_-TPR and H_2_-TPD profiles (Figure 7). Below 1% Au, the catalysts are composed mainly of Au­(I) and Au­(III) single atoms, whereas 5% Au@ceria is mainly composed of Au(0) nanoparticles but also includes Au clusters and atomically dispersed atoms (see below).^41^ In light of these results, Au­(I) appears to poison the ceria surface in 0.5% Au@ceria by decreasing its reducibility. However, further enrichment of the ceria surface with Au­(III) and Au(0) species above 1 wt % Au loading allows partial recovery of the reducibility that promotes the catalytic activity.

To further explore the surface state of the catalysts, XPS analysis was performed on the spent 1% Au@ceria catalyst (Figure S15, spectrum d). The band attributed to Au­(III) is no longer present, suggesting that Au­(III) species are either destabilized on the ceria surface orpartially transformed into Au­(I) species upon reaction. The surface molar ratio measured from the XPS spectra reveals a slightly lower density of surface Au species compared to the fresh catalyst (Au/Ce = 0.0061 for the fresh 1% Au@ceria vs Au/Ce = 0.0058 for the spent fresh 1% Au@ceria), thus supporting the ICP results. This finding is in accordance with the ICP-OES results, revealing no metal leaching. The proportion of surface-reduced cerium (Ce^3+^) is moderately enhanced to ca. 32%, confirming that the redox cycle of cerium plays a key role during the reaction (Table S3, entry 8).

The surface Au concentration and Au/Ce molar ratios were measured from XPS spectra. For all samples, the surface Au concentration ranged from 66 to 83%. The Au/Ce molar ratios increase with the Au loading from 3.2 × 10^–3^ for 0.5% Au@ceria to 37 × 10^–3^ for 5% Au@ceria (Table S3). These ratios are somewhat lower than the bulk ratios measured from ICP-OES. This observation can be explained by a homogeneous distribution of Au on the surface of elementary ceria nanoparticles in the samples without preferential enrichment on the external surface of ceria agglomerates (i.e., the sample does not behave like an infinitely thick support).

#### 3.4.7 HAADF-STEM

The Au dispersion on ceria was investigated by HAADF-STEM (EDXS) on the parent ceria and Au@ceria samples with 0.5% Au, 1% Au, and 5% Au (Figures 8 and 9). The EDX mapping of 5% Au@ceria shows a distribution of Au nanoparticles ranging from 2 to 20 nm (Figure 8a–d). In 1% Au@ceria, there are far fewer Au clusters with only occasional 1–2 nm scale clusters and a small number of particles with a diameter of 5–8 nm (Figure 8e–h). Atomic resolution imaging of 1% Au@ceria shows a possible two-dimensional Au structure (Figure 9a), indicating that Au coverage approaches single Au atom distributions. Finally, no Au nanoparticles or clusters are observed in 0.5%@ceria in any EDXS maps (Figure 8i–l).

**Figure 8:**
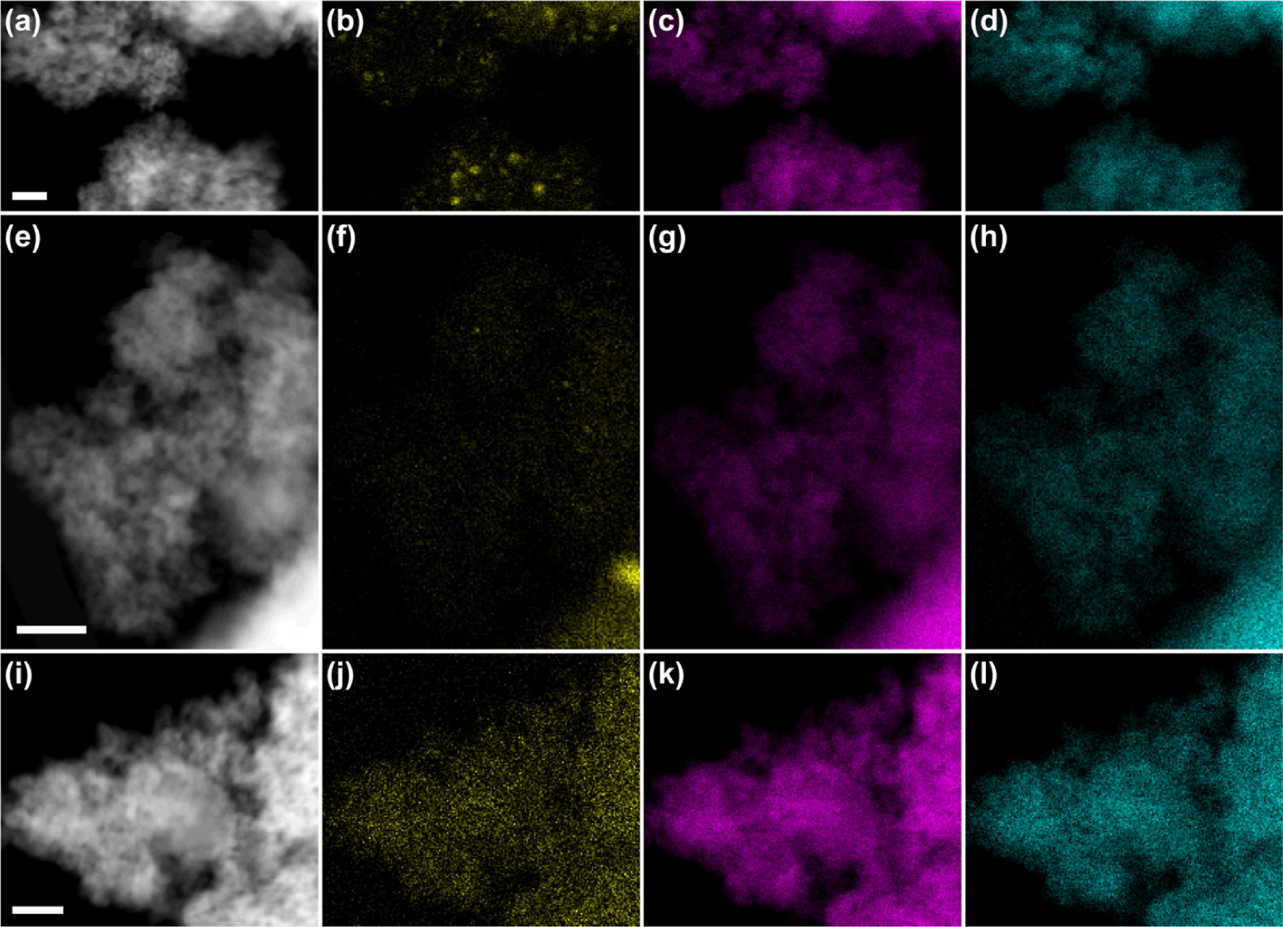
Normalized intensity EXDS elemental mapping of (a–d) 5% Au@ceria, (e–h) 1% Au@ceria and 0.5% Au@ceria, showing HAADF images (a,e,i) and Au (b,f,j), Ce (c,g,k), and O (d,h,l) signals, respectively. Scale bars are all 20 nm.

**Figure 9:**
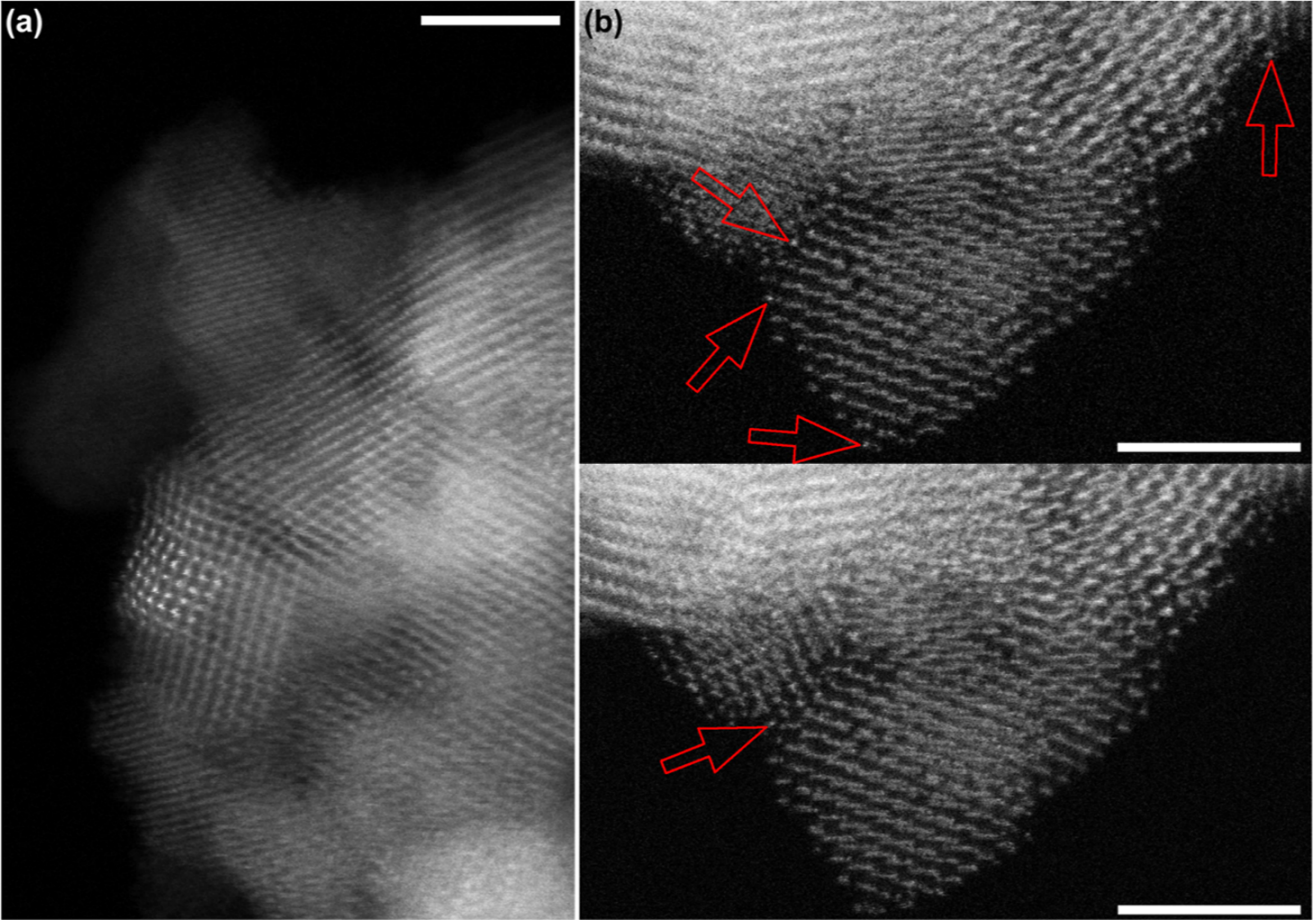
HAADF-STEM micrograph of (a) 1% Au@ceria showing a pattern of brighter atoms that is possibly a 2D “raft” of Au atoms. (b) 0.5% Au@ceria taken in sequence of single area of showing brighter atoms that are highly mobile under the electron beam and therefore are likely to be single Au atoms. Scale bars are 3 nm.

The absence of clusters in 0.5% Au@ceria implies that the Au species identified from the EDXS spectrum of 0.5% Au@ceria should be atomically dispersed (or in ultrasmall clusters). As a matter of fact, with good counting statistics, we do indeed detect a significant (above background and noise) Lα signal for Au in 0.5% Au@ceria, which is absent in the parent ceria (Figure S16) but does not correspond to any observable nanoparticles. We attribute the Au signal detected to single atoms, as evidenced from the HAADF-STEM imaging (Figure 9b). However, detection of single Au atoms on ceria is difficult due to a lack of strong contrast. Overall, these results agree well with the Au 4f XPS spectra of the Au@ceria samples (Figure S15), showing that Au at low concentration is predominantly atomically dispersed, while larger nanoparticles form at higher Au content with a higher predominance on 5% Au@ceria.

#### 3.4.8 Formation of Radical Species

##### 3.4.8.1 Nature of Radical Species and Abundance

To interrogate the formation of active radical species in our catalytic system, we carried out an EPR study using, initially, 5,5-dimethyl-1-pyrroline-*N*-oxide (DMPO) as a spin trap. The addition of short-lived O-centered radicals to nitrone or nitroso spin traps is very fast (second-order kinetic rate constants of the order of 10^9^ M^–1^·s^–1^) and produces longer-lived aminoxyl radical (also referred to as nitroxide) adducts, enabling EPR detection.^74^ As shown in Figure 10a­(i–iii), no radicals were detected when only (i) the solvent (acetonitrile); (ii) BnOH, cyclohexanone and acetonitrile; or (iii) BnOH, TBHP, cyclohexanone, and acetonitrile, were tested at the reaction conditions (60 °C for 2 min with further 2 min after adding DMPO). In contrast, when TBHP and ceria are present in the reaction medium, a signal is observed with an isotropic g-value of *g*_iso_ = 2.006 and isotropic hyperfine coupling constants (hccs) of the unpaired electron on the aminoxyl moiety of the DMPO radical adduct to the ^14^N nucleus, *a*_iso_(^14^N) = 1.36 mT, and the ^1^H^β^, *a*_iso_(^1^H^β^) = 1.06 mT [Figure 10a­(iv)]. This signal matches earlier reported values for DMPO-trapped *tert*-butyl peroxyl (*t*Bu-OO^•^) and *tert*-butyl oxyl (*t*Bu-O^•^) radicals in acetonitrile (see simulation in Figure 10b).^75^ This signal is also observed starting from BnAH instead of BnOH under the same reaction conditions, even if the amplitude is lower [Figure 10a­(v)]. Meanwhile, the spectra in Figure 10a­(iv–vi) highlight a much larger EPR signal amplitude of the DMPO radical adduct when both BnOH and BnAH are absent.

**Figure 10:**
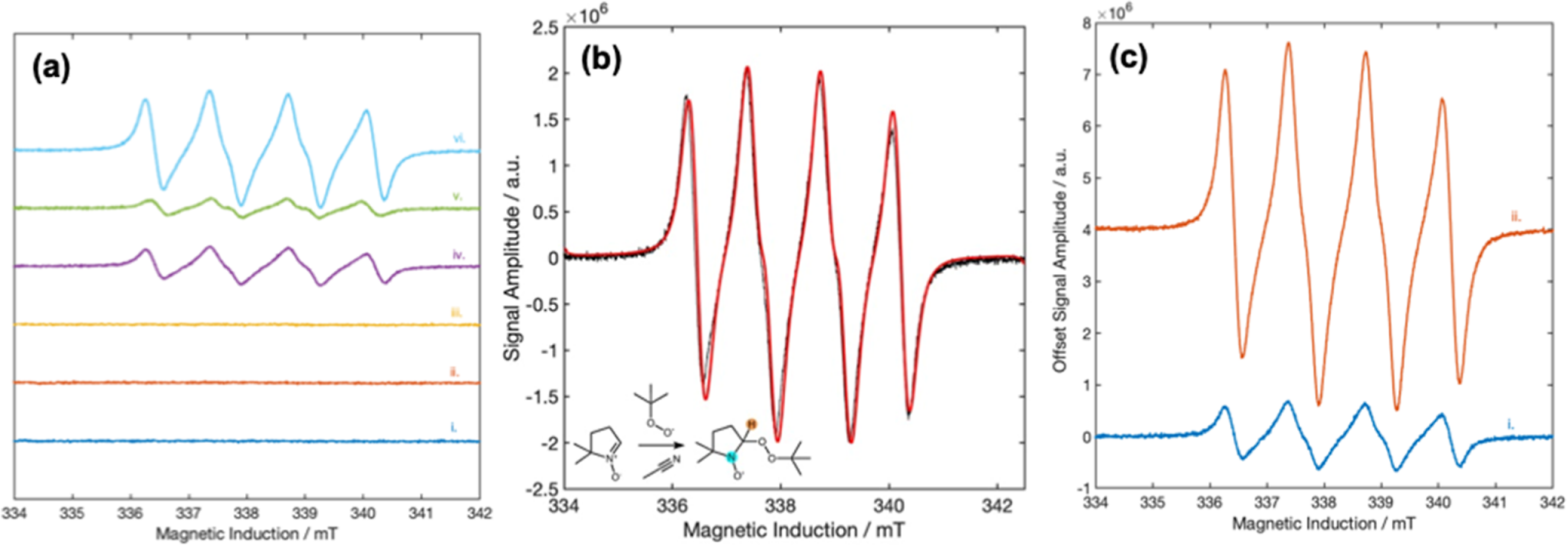
CW EPR spectra of (a­(i)) 10 mL of acetonitrile with 0.13 mmol of DMPO; (a­(ii)) 1.39 mmol of BnOH, 2 mmol of cyclohexanone, and 10 mL of acetonitrile with 0.13 mmol of DMPO; (a­(iii)) 1.39 mmol of BnOH, 2.07 mmol of TBHP, 2 mmol of cyclohexanone, and 10 mL of acetonitrile with 0.13 mmol of DMPO; (a­(iv)) 1.39 mmol of BnOH, 2.07 mmol of TBHP, 2 mmol of cyclohexanone, 10 mL of acetonitrile, and 100 mg of ceria (uncalcined) with 0.13 mmol of DMPO; (a­(v)) 1.39 mmol of BnAH, 2.07 mmol of TBHP, 2 mmol of cyclohexanone, 10 mL of acetonitrile, and 100 mg of ceria (uncalcined) with 0.13 mmol of DMPO; and (a­(vi)) 2.07 mmol of TBHP, 2 mmol of cyclohexanone, 10 mL of acetonitrile, and 100 mg of ceria (uncalcined) with 0.13 mmol of DMPO. The samples were tested at the reaction conditions (60 °C for 2 min with further 2 min after adding DMPO). (b) Experimental (black trace) and simulation (red trace) of (a­(vi)). (c) Comparison of EPR spectra of 1.39 mmol of BnOH, 2 mmol of cyclohexanone, and 10 mL of acetonitrile with 0.13 mmol of DMPO in the presence of 100 mg of ceria (uncalcined) (c­(i)) and 0.5% Au@ceria (c­(ii)).

We next measured the CW EPR spectra of BnOH and BnAH under reaction conditions in the presence of ceria but without TBHP (Figure S17a). The first spectrum (BnOH) [Figure S17a­(i)] reveals the presence of a new signal characterized by *g*_iso_ = 2.006, and hccs with *a*_iso_(^14^N) = 1.45 mT and *a*_iso_(^1^H^b^) = 2.10 mT (simulation in Figure S17b) that can be attributed to a DMPO-trapped hydroxybenzyl radical [PhCH­(OH)^•^],^74,75^ matching earlier observations.^76^ In contrast, the second spectrum (BnAH) does not show any signal. Interestingly, benzoylperoxy [PhC­(O)-OO^•^] radicals are known to be generated upon contact of BnAH with O_2_ in the presence of a radical promoter that can further drive the formation of BzOH.^76^ Formation of these radicals is discouraged when BnAH coexists with BnOH in the reaction system, even at low concentrations (2 wt %), due to the scavenging effect of [PhC­(O)-OO^•^] radicals by BnOH, resulting in the formation of stable [PhCH­(OH)^•^] radicals. The fast formation of BnAH in our reaction system in the presence of TBHP suggests, at first sight, a different radical-type mechanism proceeding either via *t*Bu-OO^•^ and *t*Bu-O^•^ radical pairs in solution (i.e., Fenton-like^77^) or via surface species stabilized on ceria.

We also investigated the formation of radical species on 0.5% Au@ceria in the presence of BnOH and TBHP under the same reaction conditions (Figure 10c). The signal is drastically magnified in the presence of Au­(I) (c­(ii)) compared to that measured for the parent ceria (uncalcined) (c­(i)). This observation contrasts with the lower catalytic activity of 0.5%@ceria compared to that of the parent ceria, which suggests that radical species in solution are not the active species of the reaction. Accordingly, the reaction is not regarded as proceeding via a Fenton-like mechanism.

Although informative on the radicals formed with/without TBHP, and when BnOH is replaced by BnAH, the DMPO experiments remain inconclusive in establishing whether, in the presence of TBHP, the radicals generated during the catalytic process are peroxyl or oxyl (or a mixture of both). This is because DMPO-*t*Bu-OO^•^ and DMPO-*t*Bu-O^•^ radical adducts have nearly identical hccs in acetonitrile,^74,78^ which cannot be distinguished due to the broadness of the spectra’s line widths. To overcome this shortcoming, we repeated the experiment with the reaction medium containing BnOH as a substrate but using another spin-trap, i.e., *N*-*tert*-butyl-a-phenylnitrone (PBN). Results with PBN are shown in Figure S18 and presented in comparison with the exact same reaction medium but with DMPO as a spin trap, Figure 10a. The spectrum in Figure S18 shows a nitrogen isotropic hyperfine coupling constant *a*_iso_(^14^N) = 1.36 mT. This value aligns more closely with the hcc of a *t*Bu-OO^•^ radical trapped by PBN [*a*_iso_(^14^N) = 1.35 mT] than with that of a *t*Bu-O^•^ radical trapped by PBN [*a*_iso_(^14^N) = 1.43 mT]. Additionally, the *a*_iso_(^1^H^b^) hccs offer limited distinction, as the values for PBN-*t*Bu-OO^•^ and PBN-*t*Bu-O^•^ radicals are 0.16 and 0.18 mT, respectively.

Overall, this body of results points out that *t*Bu-OO^•^ (peroxyl) is the main radical species present in solution and that no *t*Bu-O^•^ (oxyl) radicals are formed. This result reinforces the idea that the reaction does not proceed via a Fenton-like catalytic mechanism in solution promoted by the Ce^4+^/Ce^3+^ redox pump of ceria that should encompass the concomitant formation of *t*Bu-OO^•^ and *t*Bu-O^•^ radicals. In addition, BnOH does prevent any apparent scavenging effect of radicals issued from BnAH oxidation, pointing out that the catalytic mechanism does not encompass [PhC­(O)-OO^•^] and [PhCH­(OH)^•^] radicals in solution.^76^ In light of these results, the reaction is likely to proceed via a surface mechanism encompassing the formation of *t*Bu-OO^•^ radicals as side, nonreactive radicals in solution.

##### 3.4.8.2 Understanding Radical Adduct Abundance

Figure 10c shows completely different trends in terms of relative radical adduct concentrations when using 0.5Au@ceria and ceria catalysts, DMPO and PBN as spin traps, and BnOH and BnAH as starting reactants. In the straightforward scenario, these results demonstrate that the spin traps in such a reaction system are not simply spectator molecules “waiting” to trap radicals formed or released in solution. Their chemistry is more complex, and it is likely that they interact with the catalyst together with the substrate (BnOH or BnAH) and TBHP after addition to the reaction media in the EPR tests.

To gain understanding of the relative concentrations of radical adducts formed with different spin traps and substrates, we conducted atomistic simulations based on DFT to establish the adsorption characteristics of DMPO, PBN, and their corresponding radical adducts, on Au(111) and oxygen-terminating CeO_2_(111). All adsorption energies are listed in Table 2. DMPO adopts a perpendicular conformation over both Au(111) and CeO_2_(111) (Figure S19), with the N^+^–O^–^ bond anchored to the surface at a hollow site. In contrast, PBN adopts a loosely parallel configuration wherein the benzylic ring lies effectively parallel to the surface (5.3°), maximizing bonding interactions between the metal surface and the aromatic π-system (Figure S20). Analogous surface configurations occur for DMPO-*t*Bu-OO^•^ and PBN-*t*Bu-OO^•^ radicals over Au(111) and CeO_2_(111) (Figures S21 and S22). The interaction between DMPO and PBN over ceria is favorable (−1.04 eV and −0.93 eV), suggesting that both traps adsorb on CeO_2_(111) after addition to the reaction media. However, adsorption might be competitive against BnOH with an adsorption energy of −0.95 eV. The adsorption of DMPO-*t*Bu-OO^•^ and PBN-*t*Bu-OO^•^ radicals is also favorable on CeO_2_(111) with values of −0.81 eV and −1.15 eV, respectively. The stronger adsorption of PBN-*t*Bu-OO^•^ radicals on CeO_2_(111) compared to that of DMPO-*t*Bu-OO^•^ radicals suggests a lower concentration of the former radicals in solution, matching the EPR results after the reaction of BnOH and TBHP over ceria [Figures 10a­(iv) and S18i].

**Table 2:** Adsorption Energies (in eV) of Molecular Species on Au(111) and CeO_2_(111) Surfaces in the Gas Phase^a^

species	Au(111)	CeO_2_(111)
DMPO	–0.74 (−0.04)	–1.04
PBN	–1.51 (+0.02)	–0.93
DMPO-*t*Bu-OO^•^	–1.39 (+0.20)	–0.81
PBN-*t*Bu-OO^•^	–1.61 (+0.14)	–1.15
BnOH	–1.12 (+0.06)	–0.95
BnAH	–0.96 (+0.01)	–0.67
TBHP	–0.71 (+0.12)	–0.42
*t*BuOH	–0.66 (+0.11)	–0.87

a The additional energy contribution derived from the implicit solvation with acetonitrile is in parentheses.

To rationalize why the experiments performed with DMPO and BnAH over ceria result in a lower EPR signal compared to DMPO and BnOH [Figure 10a­(iv,v)], we computed the adsorption energies of BnAH, BnOH, TBHP, and *t*BuOH on CeO_2_(111) (Table 1). The corresponding surface configurations are represented in Figures S23–S26. BnOH adsorption is much stronger compared to BnAH (−0.95 eV vs −0.67 eV), suggesting that, upon addition of DMPO, BnOH adsorption can compete with the adsorption of free DMPO and DMPO-*t*Bu-OO^•^ radicals, resulting in a higher concentration of radicals in solution and, thus, in a more intense signal matching the experimental observation.

Finally, we conducted a series of calculations to compare the adsorption strength of DMPO/PBN and the corresponding radicals on CeO_2_(111) and Au(111). DMPO adsorption is weaker on Au(111) compared to CeO_2_(111) (−0.74 eV vs −1.04 eV), whereas the opposite trend is observed for PBN (−1.51 eV vs −0.93 eV). In addition, DMPO-*t*Bu-OO^•^ and PBN-*t*Bu-OO^•^ radicals adsorb much stronger on Au(111) than on CeO_2_(111) (−1.39 eV vs −0.81 eV and −1.61 eV vs −1.15 eV, respectively). All combined, these results suggest that in the presence of Au(111), DMPO-*t*Bu-OO^•^ and PBN-*t*Bu-OO^•^ radicals should be adsorbed on the metal with poor abundance in solution. Opposing these results, the EPR spectrum on 0.5Au@ceria [Figure 10c­(ii)] clearly shows an increased abundance of DMPO-*t*Bu-OO^•^ radicals in solution compared to ceria in the presence of BnOH and TBHP. This observation supports the lack of presence of Au^0^ on 0.5Au@ceria with the excess abundance of radicals being attributed to well-dispersed, single-site Au­(I) atoms on ceria that can favor the formation and/or desorption of *t*Bu-OO^•^ radicals during the reaction.

## 4 Conclusions

Nanoceria efficiently catalyzed the base-free, metal-free oxidation of aromatic and aliphatic alcohols, including cyclohexanol and 2-octanol, using *t*-butyl hydroperoxide as an oxidant and acetonitrile as a solvent. The catalyst achieved full conversion of benzyl alcohol into benzoic acid (60 °C, 72 h) with a specific activity of 0.24 mmol h^–1^ g^–1^ and 100% carbon balance, making nanoceria cost-competitive and greener compared to a benchmark 0.3 wt % Au/TS-1 catalyst with a 60% cost reduction and an *E*-factor of 0.08 vs 0.2–1.3 at the same benzoic acid production rate. Nanoceria was robust during the reaction and could be reused for at least five consecutive runs.

Peroxyl (*t*Bu-OO^•^) was a predominant radical generated during the reaction, with no formation of oxyl (*t*Bu-O^•^) radicals, providing evidence against a Fenton-like catalytic mechanism in solution. The radical abundance was sensitive to the spin trap used due to a divergent interaction of the trap and radical adducts with the catalyst surface. For a complete mechanism of radical adduct formation after adding the traps to reaction media in the EPR tests, it is essential to account for the coadsorption of the substrate (BnOH or BnAH), TBHP, and the spin trap on the ceria surface. Developing kinetic models that incorporate these adsorption phenomena is necessary to fully elucidate the role and involvement of spin traps in the EPR tests and potentially the full reliability of spin-trapping methodologies. Such an analysis, however, falls outside the scope of this paper and is the focus of ongoing research.

Incorporating small amounts of Au (0.5–1.0 wt %) as Au­(I) single atoms/clusters decreased the catalytic activity due to the doped ceria’s lower surface reducibility and reversible H_2_ adsorption even if more peroxyl radical species were detected in solution providing further evidence against a Fenton-like catalytic mechanism. However, the catalytic activity was partially recovered at higher Au loadings when the ceria surface was enriched with Au­(III) species and Au(0) nanoparticles, which became more reducible. Regardless of the Au loading, the product distribution remained unaffected after Au doping.

## Supplementary Material



## Notes

Catalytic performance of nanoceria for different alcohol substrates; catalytic activity of ceria and Au@ceria catalysts for BnOH oxidation; physicochemical properties, reducibility, and reversible H_2_ storage of ceria and Au@ceria catalysts; determination of activation energy for BnOH oxidation over ceria; comparison of catalytic performance of BnOH and BnAH oxidation over ceria; LC–MS chromatogram of reaction mixture; TG profiles under air of fresh and spent ceria after 5 consecutive runs; TG profiles of spent ceria after 24 h under N_2_ and air flow; N_2_ adsorption–desorption isotherms at −196 °C and BJH pore size distributions (B) of fresh and spent ceria; kinetic profiles of cinnamyl alcohol oxidation in the presence of BnOH; selectivity-conversion plots of BnAH and BzOH for BnOH oxidation using uncalcined ceria; N_2_ adsorption–desorption isotherms at −196 °C and BJH pore size distributions of ceria, ceria-400, and 5% Au@ceria; DRUV–vis spectra of Au@ceria catalysts; Ce 3d and O 1s XPS spectra of pristine ceria, 0.5% Au@ceria, 1% Au@ceria, 5% Au@ceria, and spent 1% Au@ceria after 24 h reaction; Au 4f XPS spectra of 0.5% Au@ceria (a), 1% Au@ceria (b), 5% Au@ceria (c), and spent 1% Au@ceria recovered after 24 h reaction; EDXS spectrum of pristine ceria and 0.5% Au@ceria; collection of CW EPR spectra using DMPO and PBN spin traps; BE of DMPO, DMPO-*t*Bu-OO^•^, PBN-*t*Bu-OO^•^, BnOH, BnAH, TBHP, and *t*BuOH configurations in gas phase and over Au(111) and CeO_2_(111) (PDF)
