# Supplementary material for: Nanoceria as an Efficient and Cost-Effective Metal-Free Catalyst for the Oxidation of Alcohols
Source: ACS Sustain Chem Eng. 2025 Aug 15;13(39):16348–63. doi: 10.1021/acssuschemeng.5c05061 (PMC12505376; doi:10.1021/acssuschemeng.5c05061)
Supplement: Supplementary file 1 [file sc5c05061_si_001.pdf]

Supporting Information

Nanoceria as an efficient and cost-effective metal-free catalyst for the oxidation of alcohols

Claire Squarzoni[a], Nicolas Kania[a], Malcolm Dearg[c], Max Quayle[c], Hang Hu[c], Thomas J. A. Slater[c], Andrea Folli[c], Alberto Roldan[c], Marc Pera-Titus*[b,c], Anne Ponchel*[a]

[a] Univ. Artois, CNRS, Centrale Lille, ENSCL, Univ. Lille, UMR 8181, Unité de Catalyse et de Chimie du Solide (UCCS), F-62300 Lens, France.

[b] Eco-Efficient Products and Processes Laboratory (E2P2L), UMR 3464 CNRS-Solvay, Shanghai, 201108 (P.R. China).

[c] Cardiff Catalysis Institute, School of Chemistry, Cardiff University, Main Building, Park Place, Cardiff CF10 3AT, UK.

* Corresponding author: anne.ponchel@univ-artois.fr

* Corresponding author: peratitusm@cardiff.ac.uk

TABLE CAPTIONS

Table S1. Catalytic performance of nanoceria for different alcohol substrates.[a] Page S5

Table S2. Catalytic activity of nanoceria and Au@ceria catalysts for BnOH oxidation [a] Page S6

Table S3. Main physicochemical properties of the nanoceria and Au@ceria catalysts Page S7

Table S4. Reducibility and reversible H_2 storage capacity of nanoceria and Au@ceria catalysts Page S8

Table S5. Reducibility and reversible H_2 storage capacity of nanoceria and Au@ceria catalysts Page S9

FIGURE CAPTIONS

Figure S1. TEM images of nanoceria used in this study showing particle size of *ca.* 5 nm. (A) Scale bar: 20 nm and (B) Scale bar: 10 nm. Page S10

Figure S2. Influence of the solvent on the catalytic performance of nanoceria for BnOH oxidation. Reaction conditions: 1.39 mmol of BnOH, 100 mg of nanoceria, 1.5:1 TBHP/BnOH molar ratio, 60 °C, 4 h. Page S11

Figure S3. Linear regression for the determination of the activation energy for BnOH oxidation in the presence of nanoceria. Reaction conditions: 1.39 mmol of BnOH, 100 mg of nanoceria, 1.5:1 TBHP/BnOH molar ratio, 40-80 °C. Page S12

Figure S4. Comparison of the catalytic performance of BnOH and BnAH oxidation reactions over nanoceria. Reaction conditions: 1.39 mmol of BnOH (BnAH), 100 mg of nanoceria (uncalcined), 1.5:1 TBHP/substrate molar ratio, 60 °C, 24 h. Page S13

Figure S5. (a) LC-MS chromatogram of the reaction mixture, and mass spectra of the peak at retention time (b) 9.95 min, (c) 14.92 min, and (d) 26.46 min. Reaction conditions: 1.39 mmol of BnAH, 100 mg of nanoceria (uncalcined), 1.5:1 TBHP/substrate molar ratio, 10 mL of acetonitrile, 60 °C, 24 h. Page S14

Figure S6. TG profiles under air of the fresh (black solid line) and spent (green solid line) nanoceria after 5 consecutive runs in the BnOH oxidation at 60 °C. Page S15

Figure S7. TG profiles of the spent nanoceria recovered after 24 h in the BnOH oxidation at 60 °C under N_2 flow (black solid line) and under air flow (red solid line). Page S16

Figure S8. N_2 adsorption-desorption isotherms (A) at -196 °C (filled symbols and empty symbols refer to the adsorption branch and desorption branch respectively) and corresponding BJH pore size distributions (B) of the following samples: Fresh nanoceria (green line) and nanoceria after BnOH oxidation test at 60°C. Page S17

Figure S9. Kinetic profiles of cinnamyl alcohol oxidation in the presence of BnOH. Reaction conditions: 1.39 mmol of cinnamyl alcohol, 100 mg of nanoceria, 1.5:1 TBHP/substrate molar ratio, 60 °C. Page S18

Figure S10. Selectivity-conversion plots of benzaldehyde and benzoic acid for BnOH oxidation using uncalcined nanoceria. Experimental data for the calcined nanoceria (Ceria-400) are also included for comparison. Reaction conditions: 1.39 mmol of BnOH, 100 mg of nanoceria, 1.5:1 TBHP/BnOH molar ratio, 60 °C. Page S19

Figure S11. N_2 adsorption-desorption isotherms (A) at -196 °C (filled symbols and empty symbols refer to the adsorption branch and desorption branch respectively) and corresponding BJH pore size distributions (B) of the following samples: Nanoceria (A1, B1), Ceria-400 (A2, B2), and 5%Au@ceria (A3, B3). Page S20

Figure S12. DRUV-vis absorption spectra of Au@ceria catalysts. Page S21

Figure S13. Ce 3d (*left*) and O 1s (*right*) XPS spectra of parent ceria (a and f), 0.5%Au@ceria (b and g), 1%Au@ceria (c and h), 5%Au@ceria (d and i) and spent 1%Au@ceria recovered after 24 h reaction (e and j). The Ce 3d XPS region (*left*) can contain 10 bands originated from different Ce oxidation states (Ce^{3+} and Ce^{4+}) and their 4f configurations. The pairs u_o-v_o and u'-v' are assigned to Ce^{3+} (blue lines). For the O 1s level (*right*), colored lines represent the different component bands obtained after deconvolution (olive for O 1s-A, red for O 1s-B, violet for O 1s-C and yellow for O 1s-D). Page S22

Figure S14. Ce 3d XPS spectra of (a) pristine nanoceria, (b) used nanoceria after a 24 h time-on-stream and (c) used & calcined ceria. The pairs u_o-v_o and u'-v' are assigned to Ce^{3+} (blue lines). Page S23

Figure S15. Au 4f XPS spectra of 0.5%Au@ceria (a), 1%Au@ceria (b), 5%Au@ceria (c) and spent 1%Au@ceria recovered after 24 h reaction (d). Page S24

Figure S16. EDXS spectrum of (a) parent nanoceria and (b) 0.5%Au@ceria. Page S25

Figure S17. CW EPR spectra of **(a-i)** 1.39 mmol of BnOH, 2 mmol of cyclohexanone, 10 mL of acetonitrile with 0.13 mmol of DMPO in the presence of 100 mg of nanoceria (uncalcined); **(a-ii)** 1.39 mmol of BnAH, 2 mmol of cyclohexanone, 10 mL of acetonitrile with 0.13 mmol of DMPO in the presence of 100 mg of nanoceria (uncalcined). The samples were tested at the reaction conditions (60 °C for 2 min with further 2 min after adding DMPO). **(b)** Experimental (black trace) and simulation (red trace) of **(a-i)**. Page S26

Figure S18. CW EPR spectra of **(i)** 1.39 mmol of BnOH, 2.07 mmol of TBHP, 2 mmol of cyclohexanone, 10 mL of acetonitrile, 100 mg of nanoceria (uncalcined) with 0.13 mmol of PBN; **(ii)** 1.39 mmol of BnAH, 2.07 mmol of TBHP, 2 mmol of cyclohexanone, 10 mL of acetonitrile, 100 mg of nanoceria (uncalcined) with 0.13 mmol of PBN. Page S27

Figure S19. DMPO configurations in (a) gas phase and over (b) Au(111) and (c) CeO_2(111). Page S28

Figure S20. DMPO-tBu-OO• configurations in (a) gas phase and over (b) Au(111) and (c) CeO_2(111). Page S29

Figure S21. PBN configurations in (a) gas phase and over (b) Au(111) and (c) CeO_2(111). Page S30

Figure S22. PBN-tBu-OO• configurations in (a) gas phase and over (b) Au(111) and (c) CeO_2(111). Page S31

Figure S23. BnOH configurations in (a) gas phase and over (b) Au(111) and (c) CeO_2(111). Page S32

Figure S24. BnAH configurations in (a) gas phase and over (b) Au(111) and (c) CeO_2(111). Page S32

Figure S25. Binding energies for TBHP configurations in (a) gas phase and over (b) Au(111) and (c) CeO_2(111). Page S33

Figure S26. Binding energies for tBuOH onfigurations in (a) gas phase and over (b) Au(111) and (c) CeO_2(111). Page S33

Table S1. Catalytic performance of nanoceria for different alcohol substrates.[a]

Entry	Substrate	Conv. (%)	Yield aldehyde / ketone (%)	Yield acid (%)	Carbon loss (%)	Intrinsic activity (μmol·m^{-2}·h^{-1})	TOF (h^{-1})
1	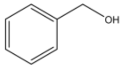 *BnOH*	69	36	32	1	1.1	1.1
2	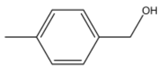 *4-methylbenzyl alcohol*	65	29	25	11	1.1	1.1
3	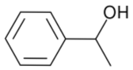 *Phenylethanol*	58	58	/	0	0.81	0.80
4	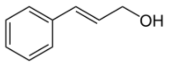 *CnOH*	67	28	1	20	1.4	1.4
5[b]	*CnOH*	69	28	2	14	1.4	1.4
6	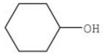 *Cyclohexanol*	36	35	/	1	0.50	0.49
7	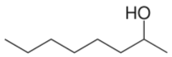 *2-octanol*	26	26	/	0	0.34	0.33

[a] Reaction conditions: 1.39 mmol of alcohol substrate, 100 mg of catalyst, 1.5:1 TBHP/alcohol molar ratio, 60 °C, 24 h.

[b] Test carried out in the presence of BnOH (1.39 mmol)

Table S2. Catalytic activity of nanoceria and Au@ceria catalysts for BnOH oxidation [a]

Entry	Catalyst	BnOH conversion (%)	Selectivity (%)		Yield (%)		Carbon loss (%)	Intrinsic activity (μmol·m^{-2}·h^{-1})
			BnAH	BzOH	BnAH	BzOH		
1	Nanoceria	69	53	46	36	32	1	1.1
2	Ceria-400	52	74	26	35	13	3	0.74
3	0.25%Au@ceria	33	86	14	28	5	0	0.49
4	0.5%@ceria	30	93	7	27	2	1	0.28
5	1%Au@ceria	37	89	11	30	4	3	0.70
6	2%Au@ceria	36	87	13	30	5	1	0.75
7	5%Au@ceria	48	82	18	38	8	1	1.1
8	2%Au-TiO_2	29	89	11	26	3	3	11

[a] Reaction conditions: 1.39 mmol of BnOH, 100 mg of catalyst, 1.5:1 TBHP/BnOH molar ratio, 60 °C, 24 h.

Table S3. Main physicochemical properties of the nanoceria and Au@ceria catalysts

Entry	Catalyst	CeO_2 crystallite size (nm)[a]	BET SSA ($m^2 \cdot g^{-1}$)[b]	Bulk analysis (ICP)		Surface analysis (XPS)			
				Au (wt%)[e]	Au/Ce (x10^3 molar)	Au/Ce (x10^3 molar)	% Au surface [f]	Au surface species	% Ce^{3+} surface [g]
1	Nanoceria	4.3	219	-	-	-	-	-	22.0
2	Ceria-400	4.7	208	-	-	-	-	-	22.1
3	0.25%Au@ceria	4.4	224	0.28	2.4	N.A.[h]	N.A.	N.A.	N.A.
4	0.5%Au@ceria	4.3	232	0.54	4.7	3.2	68	Au^{+}	18.2
5	1%Au@ceria	4.4	213	0.93	8.2	6.1	74	Au^{+}, Au^{3+}	23.7
6	2%Au@ceria	4.3	205	2.09	19	N.A.	N.A.	N.A.	N.A.
7	5%Au@ceria	4.4	213	4.28	44	37	95	Au^{0}, Au^{+}, Au^{3+}	23.5
8	1%Au@ceria (spent)[i]	NA	NA	0.86	7.6	5.8	76	Au^{+}	31.6

[a] Measured from XRD using the Scherrer equation applied to CeO_2 (111) reflection.

[b] Specific surface area calculated from the BET equation in the P/P° range of 0.05-0.30.

[c] Total pore volume estimated at P/P°=0.995.

[d] Average pore size estimated from the Barrett–Joyner–Halenda (BJH) method.

[e] Measured from ICP analysis.

[f] Calculated by dividing the XPS surface molar ratio Au/Ce by the ICP bulk molar ratio Au/Ce.

[g] Estimated by fitting using CasaXPS (see Experimental section, Characterization techniques 2.3).

[h] N.A. for Not Analyzed.

[i] 1%Au@ceria catalyst recovered after being used to oxidize BnOH in acetonitrile (60 °C, 24 h).

Table S4. Reducibility and reversible H_2 storage capacity of nanoceria and Au@ceria catalysts

Entry	Catalyst	Catalyst reducibility [a]			H_2 reversibility [a]			Ce(III)-OH (groups/nm^2)	O vacancies (groups/nm^2)
		H_2-TPR bands (°C)	H_2 uptake (mmol·g)[c]	Reducibility (%)	Main H_2-TPD bands (°C)	H_2 release (mmol·g)[c]	Reversible H_2 (%)		
1	Nanoceria	445, 535	1.42 (6.5)	49	56, 357, 478	0.11 (0.51)	7.0	0.61	3.61
2	Ceria-400	440, 530	1.22 (5.9)	42	53, 362, 491	0.06 (0.29)	4.9	0.35	3.38
3	0.25%Au@ceria	180	1.08 (4.8)	37	167, 340, 509, 632, 748	0.74 (3.3)	69	3.98	0.90
4	0.5%Au@ceria	160	1.10 (4.7)	38	168, 313, 520, 566, 753	0.75 (3.2)	68	3.85	0.90
5	1%Au@ceria	135	1.13 (5.3)	39	147, 250, 384, 521, 648, 762	0.76 (3.6)	67	4.34	1.02
6	2%Au@ceria	105	1.17 (5.7)	40	101, 164, 251, 376, 506, 623, 747	0.76 (3.7)	65	4.46	1.20
7	5%Au@ceria	60	1.30 (7.1)	45	94, 148, 215, 368, 500, 628, 748	0.76 (4.1)	58	4.94	1.81

[a] Measured from the H_2-TPR profiles in the temperature range from 30 °C to 200 °C under H_2 flow [40 mL(STP)·min^{-1}]; the reducibility was measured from the H_2 uptake using Eq 2; in parentheses, data in μmol·m^{-2}

[b] Measured from the H_2-TPD profiles in the temperature range from 40 °C to 400 °C after reduction at 180 °C for 30 min using a heating ramp of 10 °C·min^{-1}

[c] H_2 release measured from the desorbed H_2 amount using Eq 3; in parentheses, data in μmol·m^{-2}

Table S5. Proportion of different species in the nanoceria samples catalysts measured by XPS.[a]

Sample	% Ce^{3+} Surf.	(O/Ce)	Concentration (mol%)[b]					(O_D/O_A)
			Ce 4d	O 1s (A) *529 eV*	O 1s (B) *531 eV*	O 1s (C) *532 eV*	O 1s (D) *533 eV*	
Ceria	22.0	2.11	32.23	36.02	6.34	19.27	6.14	0.17
Used ceria	21.0	2.32	30.23	37.68	4.21	18.11	9.77	0.26
Used & calcined ceria	24.9	1.99	33.65	43.22	1.99	21.14	0	0

[a] Estimated by fitting using CasaXPS (see Experimental section, Characterization techniques 2.3).

[b] Carbon has been omitted for clarity.

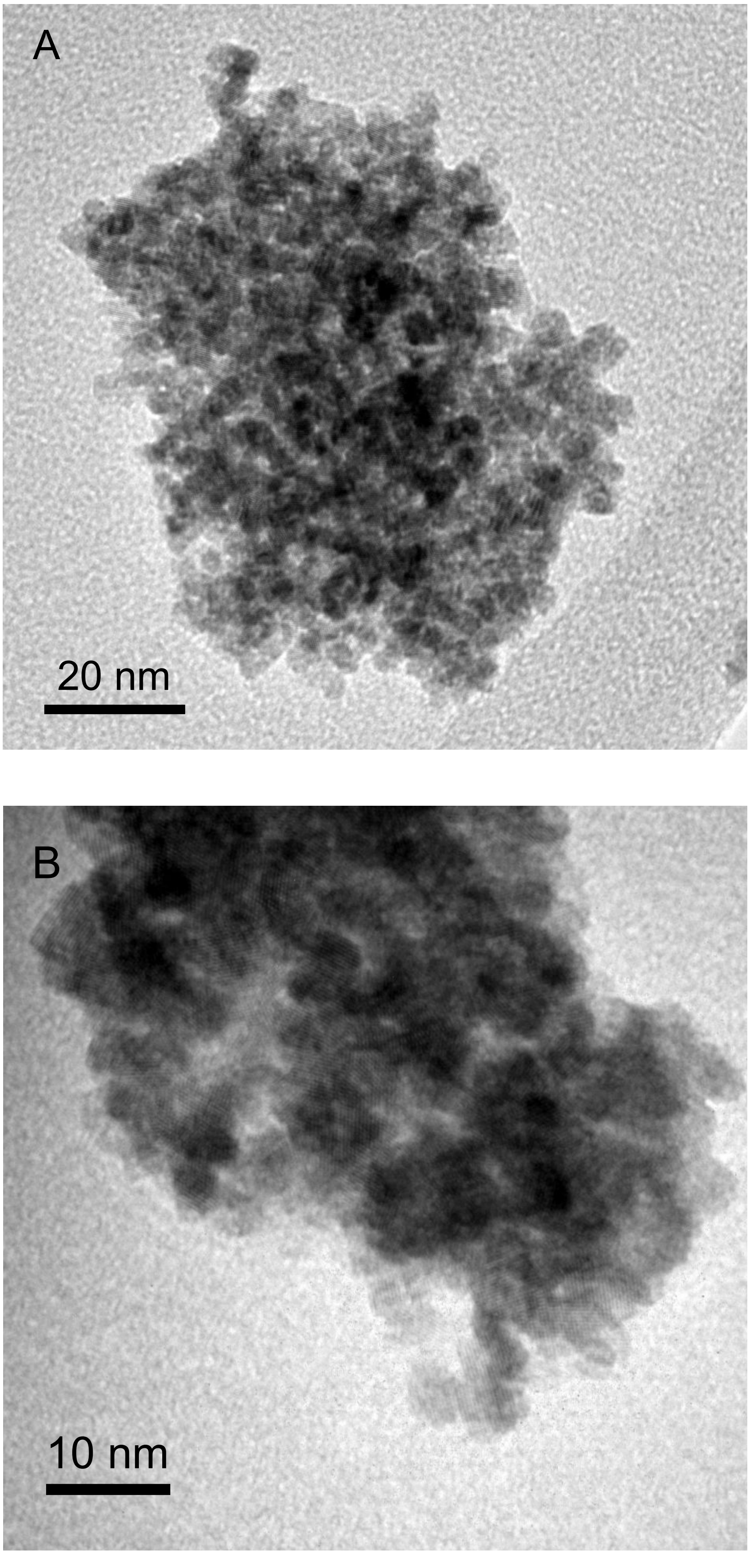

Figure S1. TEM images of nanoceria used in this study showing particle size of *ca.* 5 nm. (A) Scale bar: 20 nm and (B) Scale bar: 10 nm.

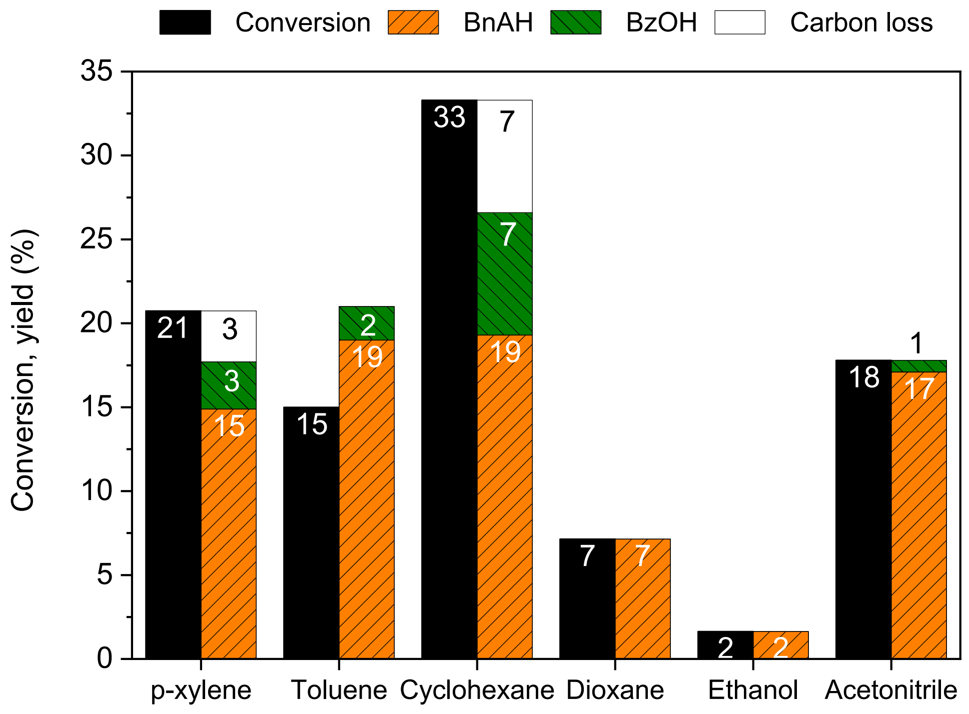

Figure S2. Influence of the solvent on the catalytic performance of nanoceria for BnOH oxidation. Reaction conditions: 1.39 mmol of BnOH, 100 mg of nanoceria, 1.5:1 TBHP/BnOH molar ratio, 60 °C, 4 h.

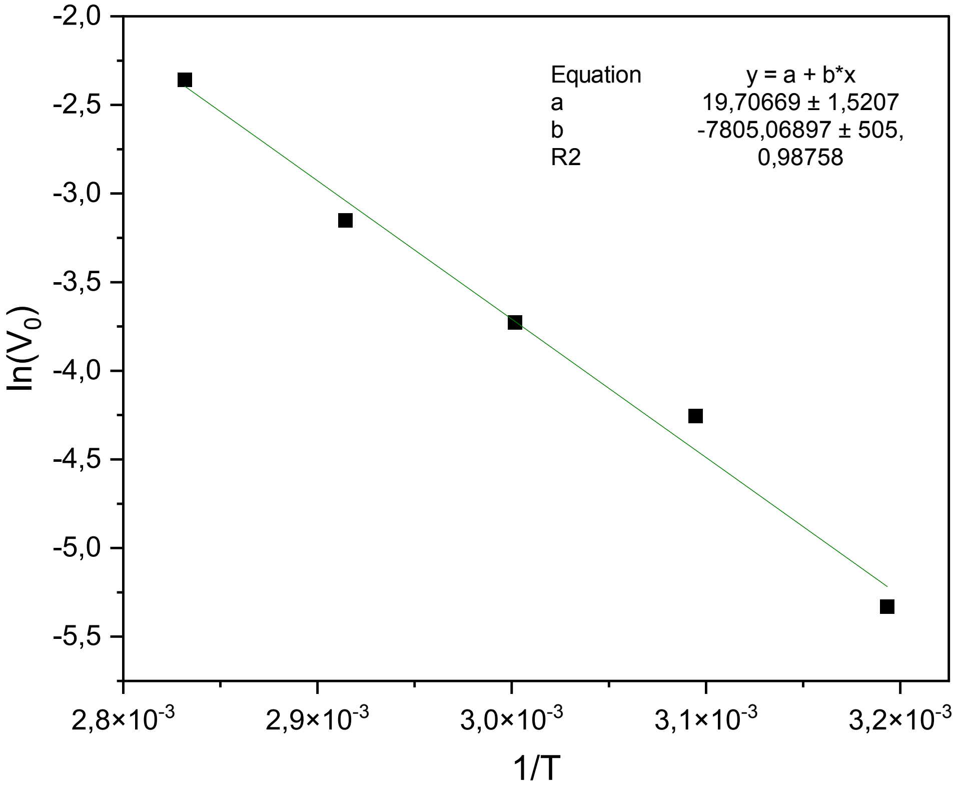

Figure S3. Linear regression for the determination of the activation energy for BnOH oxidation in the presence of nanoceria. Reaction conditions: 1.39 mmol of BnOH, 100 mg of nanoceria, 1.5:1 TBHP/BnOH molar ratio, 40-80 °C.

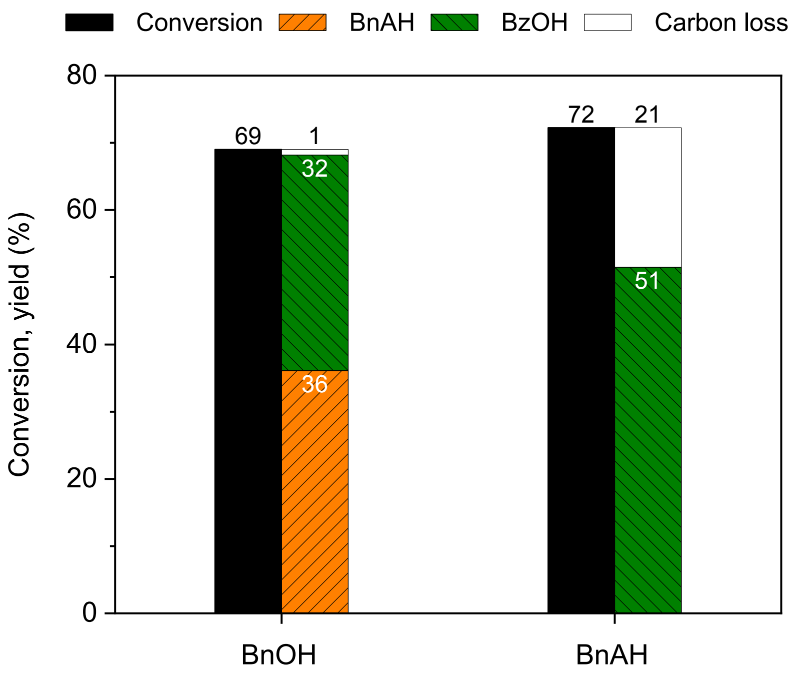

Figure S4. Comparison of the catalytic performance of BnOH and BnAH oxidation reactions over nanoceria. Reaction conditions: 1.39 mmol of BnOH (BnAH), 100 mg of nanoceria (uncalcined), 1.5:1 TBHP/substrate molar ratio, 60 °C, 24 h.

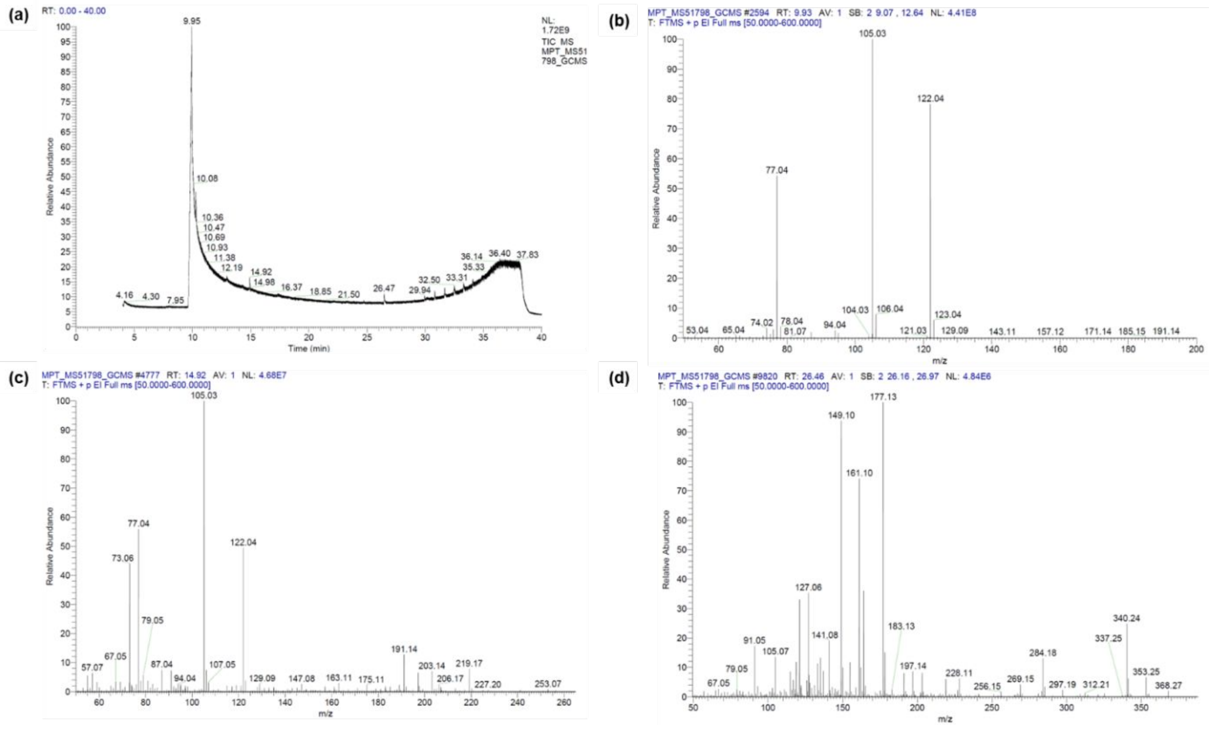

Figure S5. (a) LC-MS chromatogram of the reaction mixture, and mass spectra of the peak at retention time (b) 9.95 min, (c) 14.92 min, and (d) 26.46 min. Reaction conditions: 1.39 mmol of BnAH, 100 mg of nanoceria (uncalcined), 1.5:1 TBHP/substrate molar ratio, 10 mL of acetonitrile, 60 °C, 24 h.

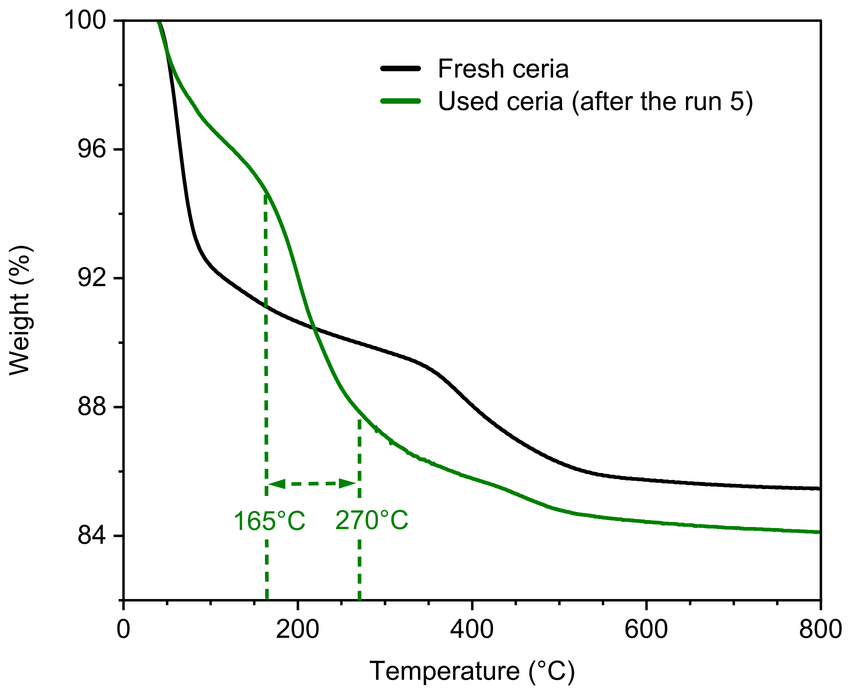

Figure S6. TG profiles under air of the fresh (black solid line) and spent (green solid line) nanoceria after 5 consecutive runs in the BnOH oxidation at 60 °C.

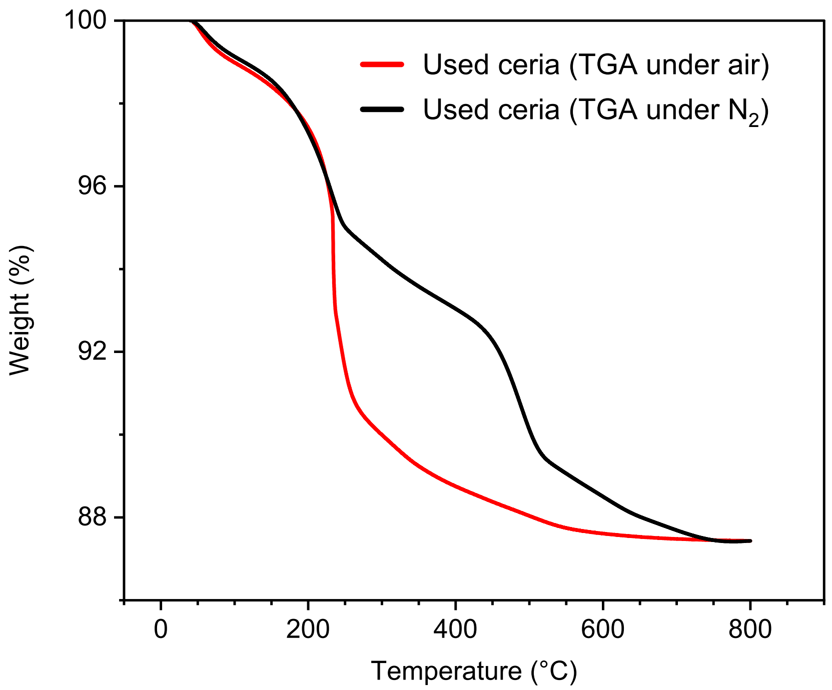

Figure S7. TG profiles of the spent nanoceria recovered after 24 h in the BnOH oxidation at 60 °C under N_2 flow (black solid line) and under air flow (red solid line).

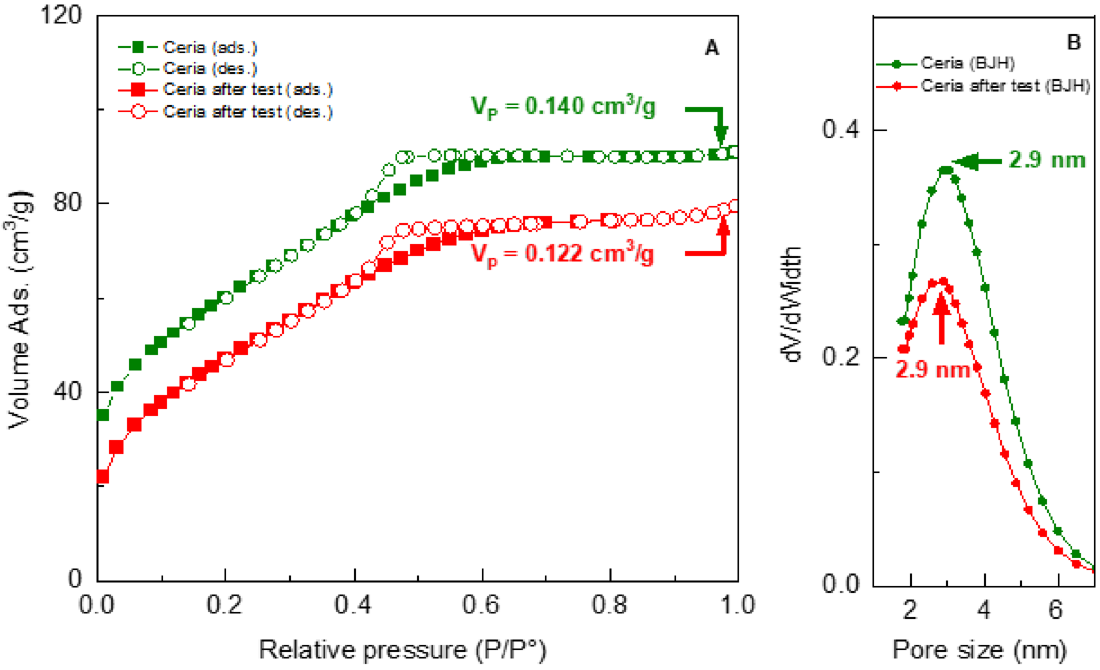

Figure S8. N_2 adsorption-desorption isotherms (A) at -196 °C (filled symbols and empty symbols refer to the adsorption branch and desorption branch respectively) and corresponding BJH pore size distributions (B) of the following samples: Fresh nanoceria (green line) and nanoceria after BnOH oxidation test at 60°C.

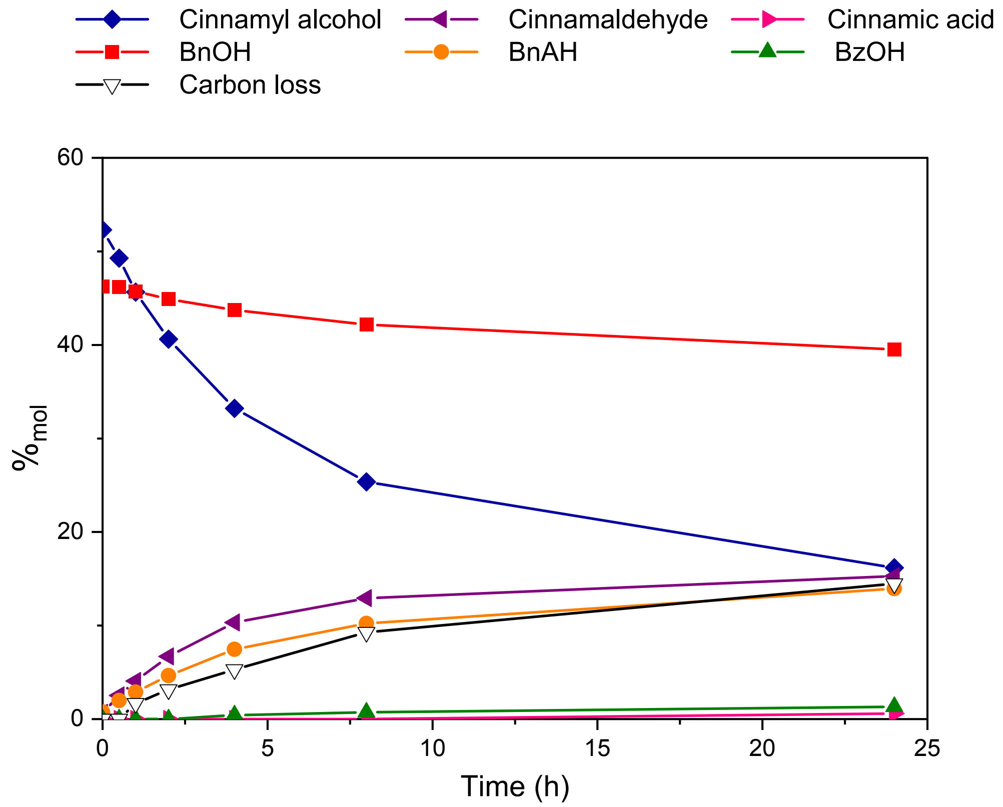

Figure S9. Kinetic profiles of cinnamyl alcohol oxidation in the presence of BnOH. Reaction conditions: 1.39 mmol of cinnamyl alcohol, 100 mg of nanoceria, 1.5:1 TBHP/substrate molar ratio, 60 °C.

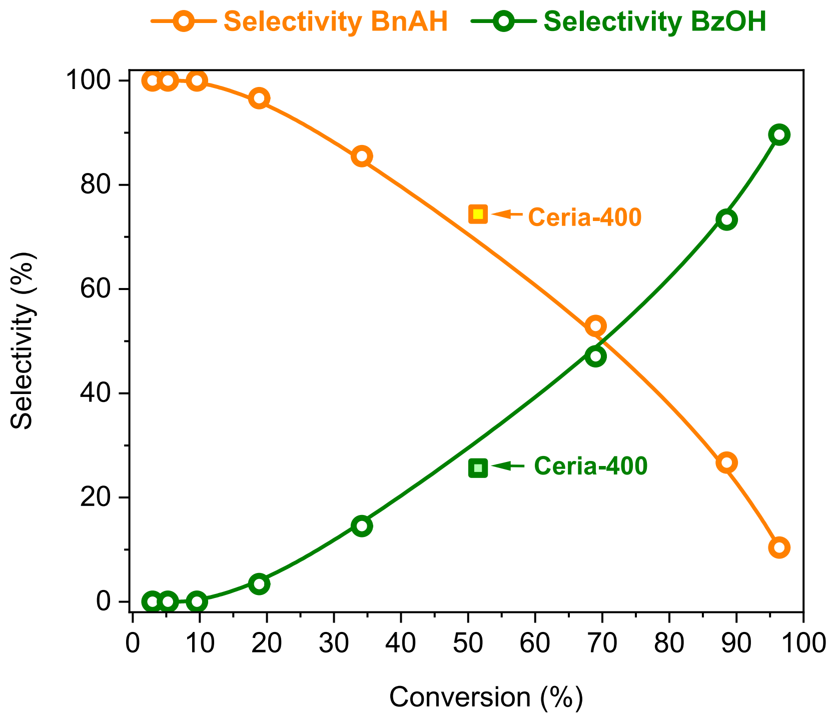

Figure S10. Selectivity-conversion plots of benzaldehyde and benzoic acid for BnOH oxidation using uncalcined nanoceria. Experimental data for the calcined nanoceria (Ceria-400) are also included for comparison. Reaction conditions: 1.39 mmol of BnOH, 100 mg of nanoceria, 1.5:1 TBHP/BnOH molar ratio, 60 °C.

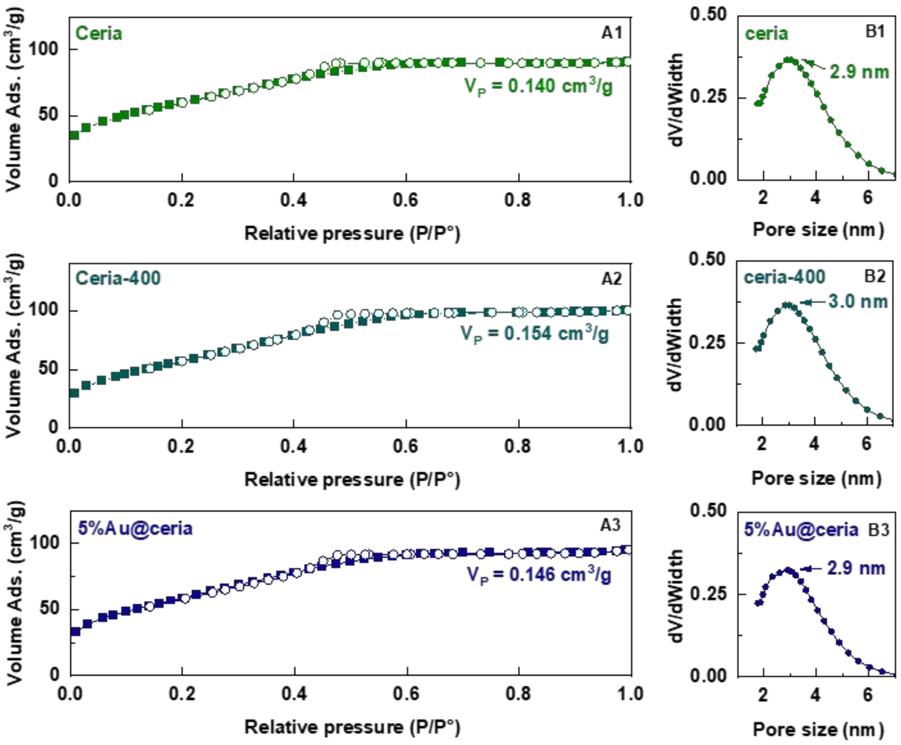

Figure S11. N_2 adsorption-desorption isotherms (A) at -196 °C (filled symbols and empty symbols refer to the adsorption branch and desorption branch respectively) and corresponding BJH pore size distributions (B) of the following samples: Nanoceria (A1, B1), Ceria-400 (A2, B2), and 5%Au@ceria (A3, B3).

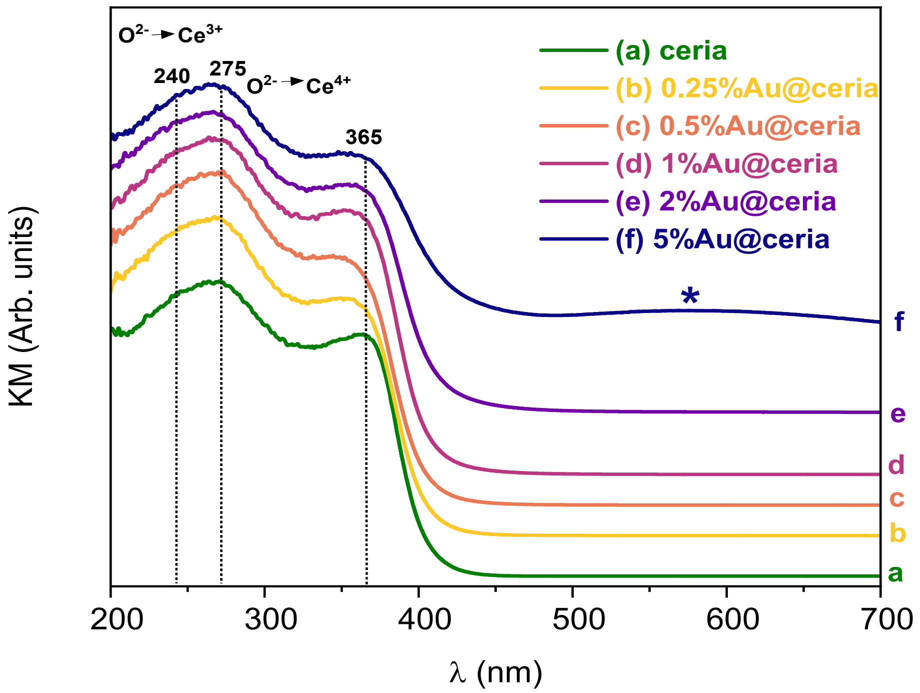

Figure S12. DRUV-vis absorption spectra of Au@ceria catalysts.

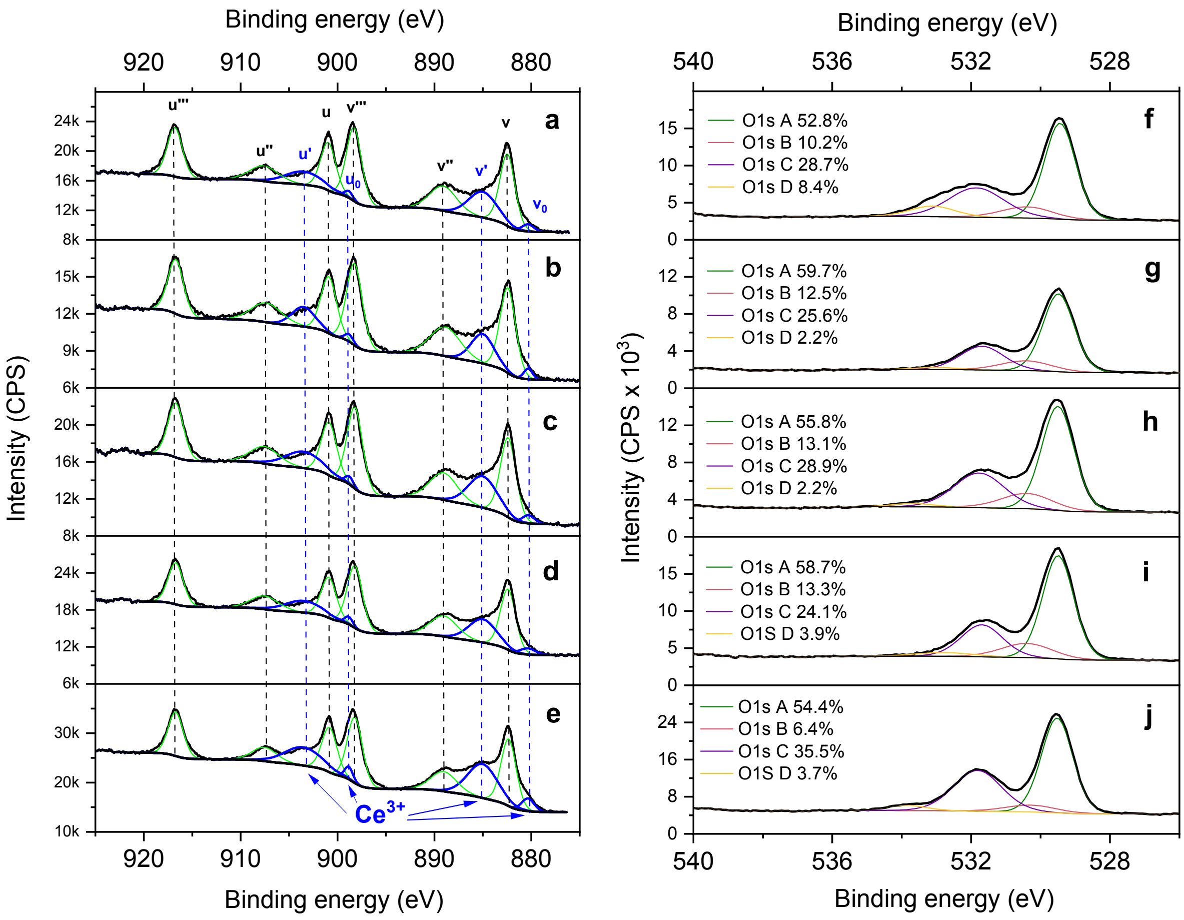

Figure S13. Ce 3d (*left*) and O 1s *(right)* XPS spectra of parent nanoceria (a and f), 0.5%Au@ceria (b and g), 1%Au@ceria (c and h), 5%Au@ceria (d and i) and spent 1%Au@ceria recovered after 24 h reaction (e and j). The Ce 3d XPS region (*left*) can contain 10 bands originated from different Ce oxidation states (Ce^{3+} and Ce^{4+}) and their 4f configurations. The pairs u_o-v_o and u'-v' are assigned to Ce^{3+} (blue lines). For the O 1s level (*right*), colored lines represent the different component bands obtained after deconvolution (olive for O 1s-A, red for O 1s-B, violet for O 1s-C and yellow for O 1s-D).

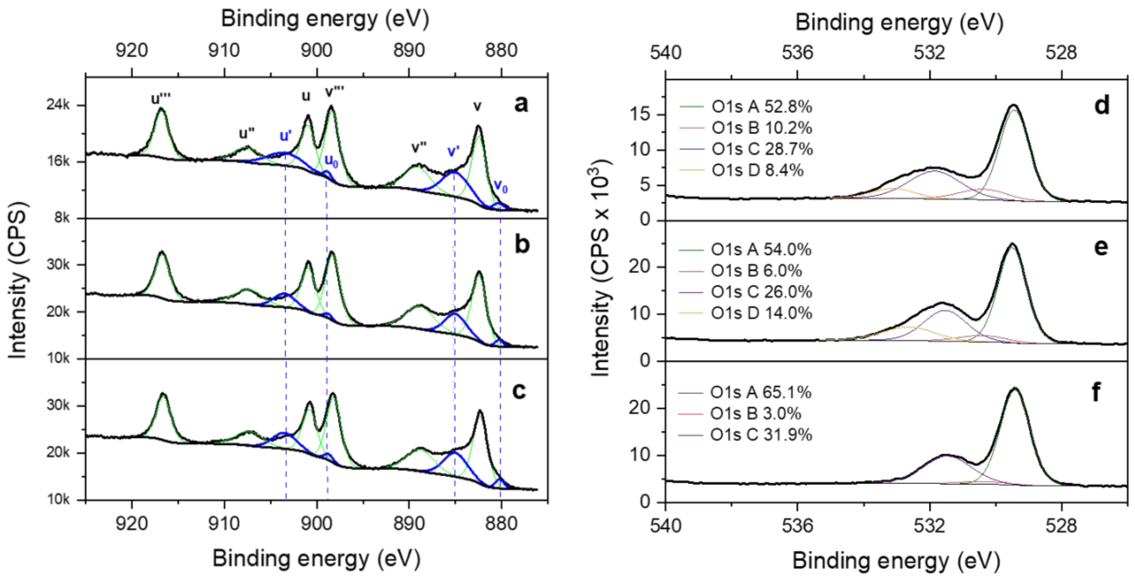

Figure S14. Ce 3d (*left*) and O 1s *(right*) XPS spectra of (a and d) pristine nanoceria, (b and e) used nanoceria after a 24 h time-on-stream and (c and f) used & calcined ceria. The pairs u_o-v_o and u'-v' *(left)* are assigned to Ce^{3+} (blue lines). The O 1s components (*right*) obtained after deconvolution are represented in olive green for O 1s-A, red for O 1s-B, purple for O 1s-C and yellow for O 1s-D.

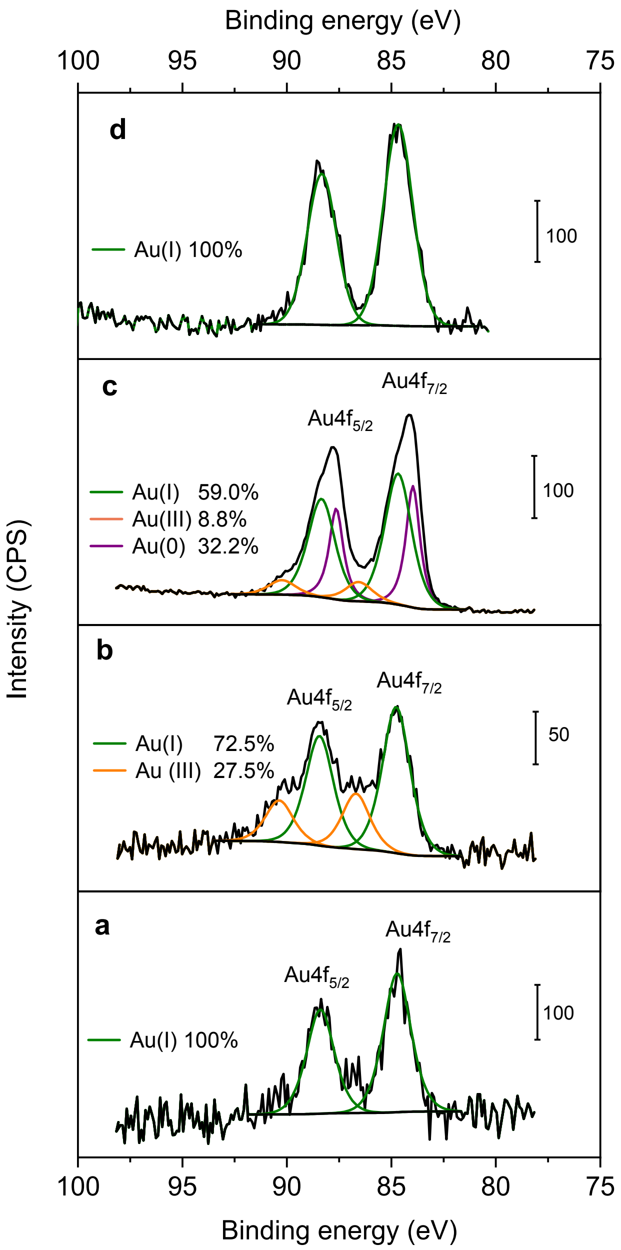

Figure S15. Au 4f XPS spectra of 0.5%Au@ceria (a), 1%Au@ceria (b), 5%Au@ceria (c) and spent 1%Au@ceria recovered after 24 h reaction (d).

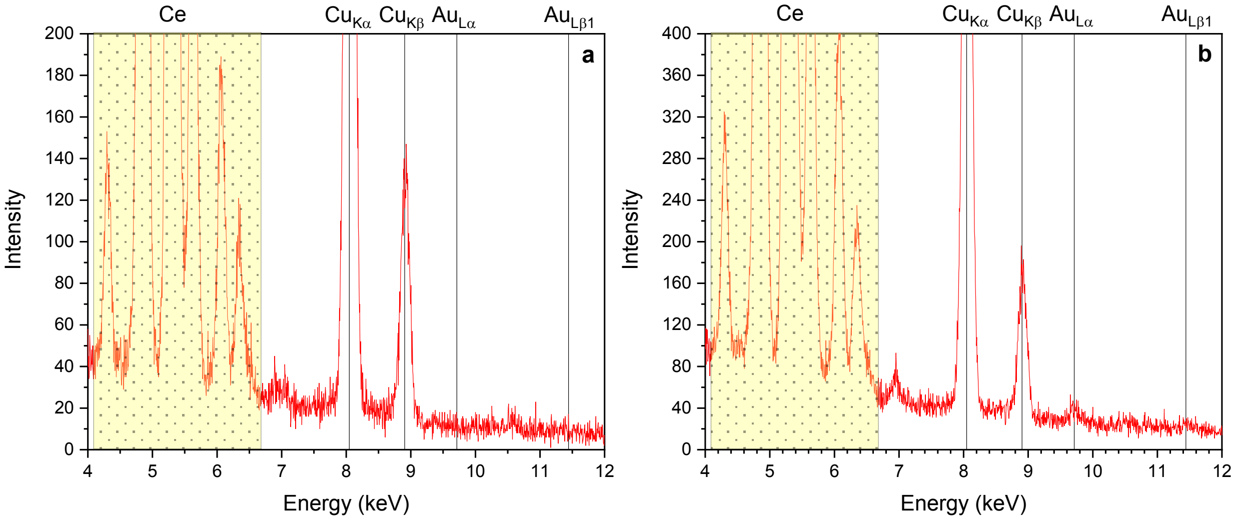

Figure S16. EDXS spectrum of (a) parent nanoceria and (b) 0.5%Au@ceria.

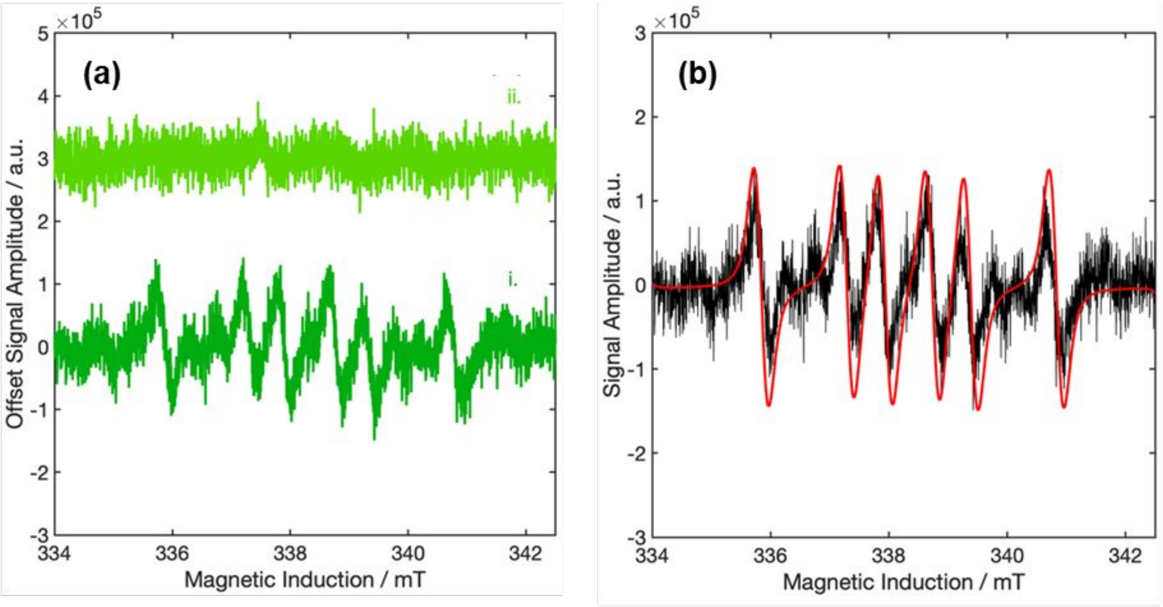

Figure S17. CW EPR spectra of **(a-i)** 1.39 mmol of BnOH, 2 mmol of cyclohexanone, 10 mL of acetonitrile with 0.13 mmol of DMPO in the presence of 100 mg of nanoceria (uncalcined); **(a-ii)** 1.39 mmol of BnAH, 2 mmol of cyclohexanone, 10 mL of acetonitrile with 0.13 mmol of DMPO in the presence of 100 mg of nanoceria (uncalcined). The samples were tested at the reaction conditions (60 °C for 2 min with further 2 min after adding DMPO). **(b)** Experimental (black trace) and simulation (red trace) of **(a-i)**.

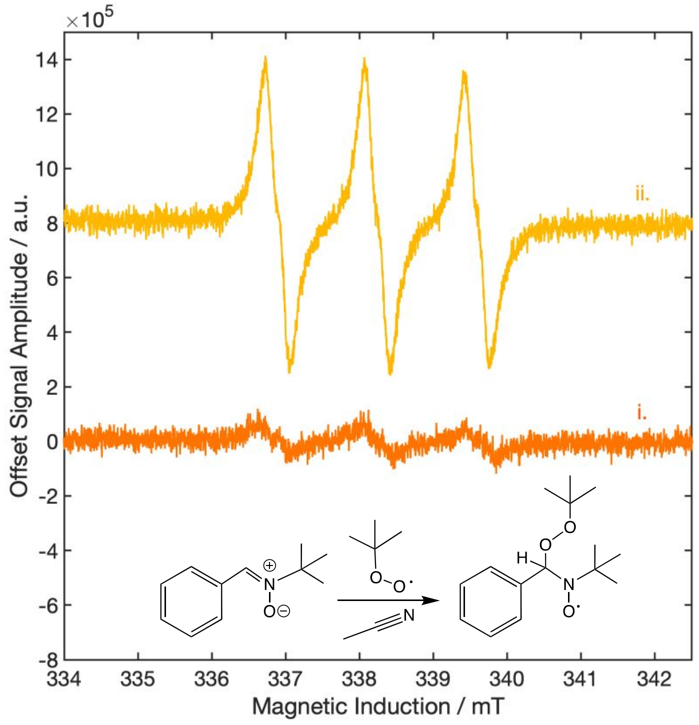

Figure S18. CW EPR spectra of **(i)** 1.39 mmol of BnOH, 2.07 mmol of TBHP, 2 mmol of cyclohexanone, 10 mL of acetonitrile, 100 mg of ceria (uncalcined) with 0.13 mmol of PBN; **(ii)** 1.39 mmol of BnAH, 2.07 mmol of TBHP, 2 mmol of cyclohexanone, 10 mL of acetonitrile, 100 mg of nanoceria (uncalcined) with 0.13 mmol of PBN.

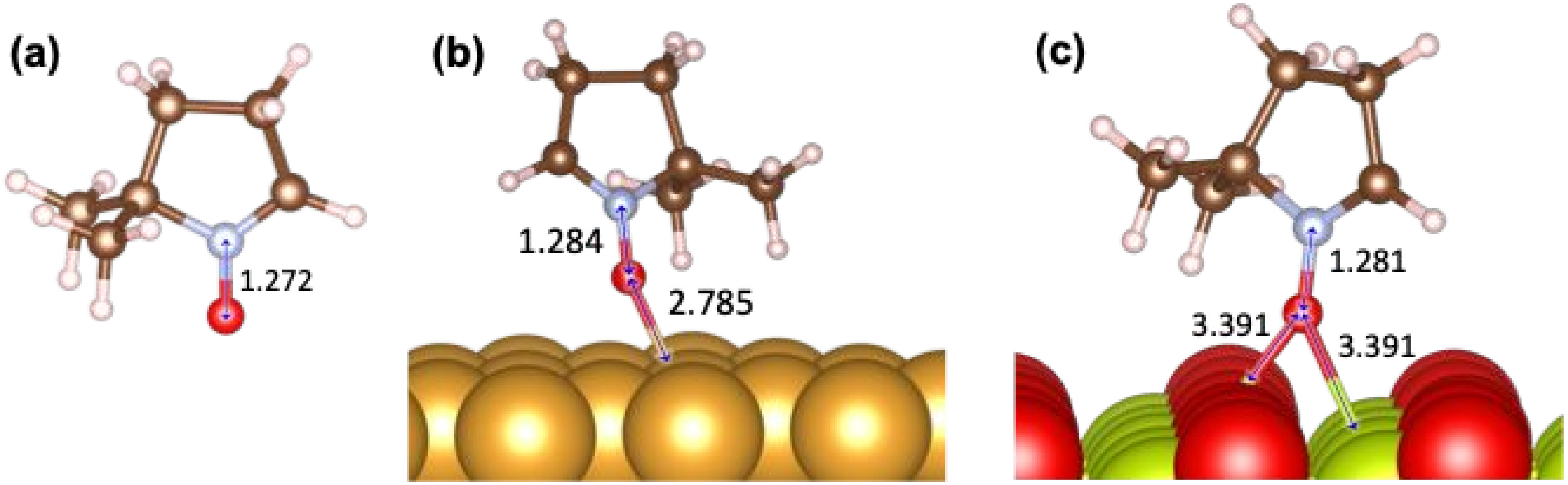

Figure S19. DMPO configurations in (a) gas phase and over (b) Au(111) and (c) CeO_2(111).

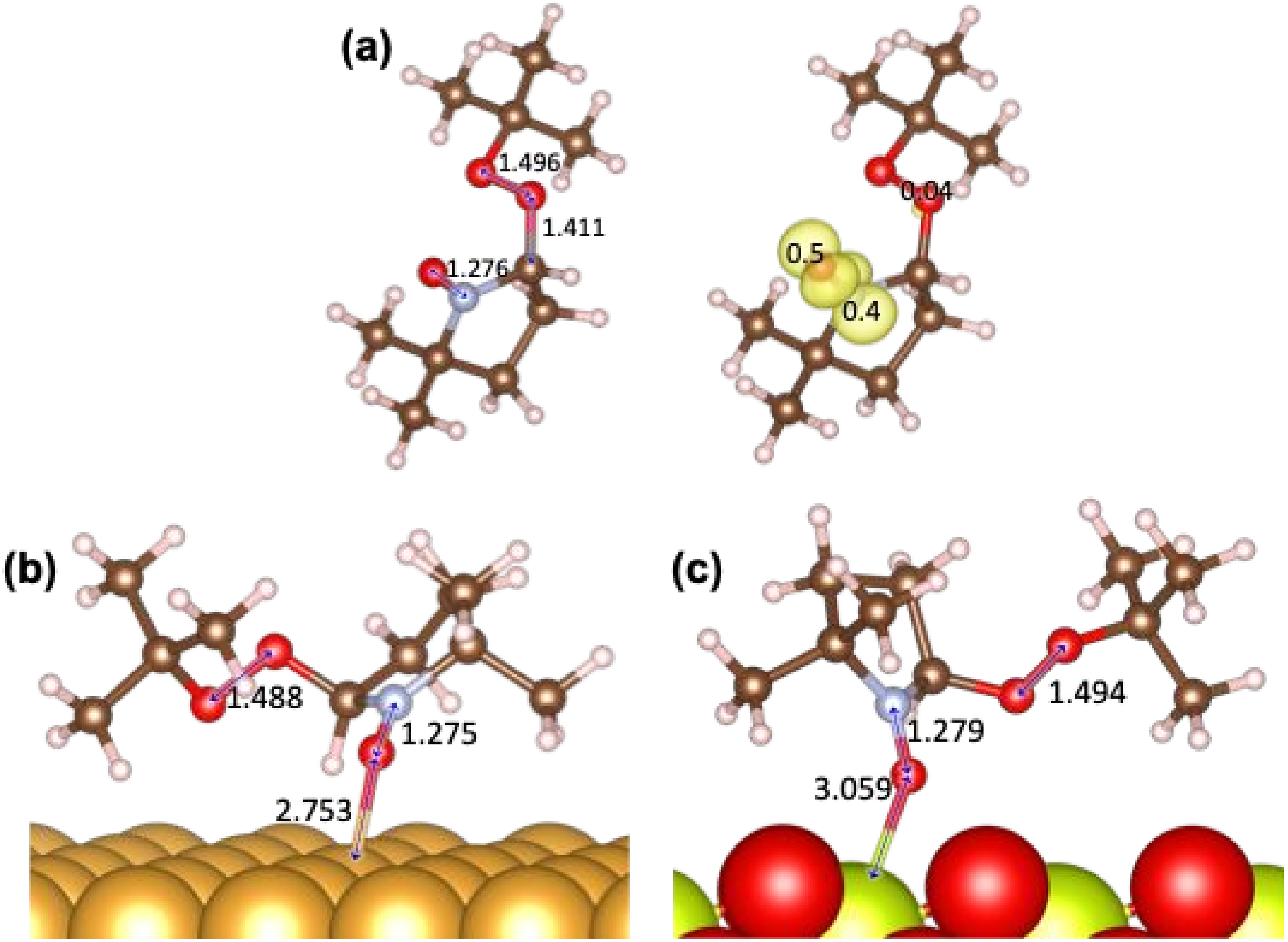

Figure S20. DMPO-tBu-OO• configurations in (a) gas phase and over (b) Au(111) and (c) CeO_2(111).

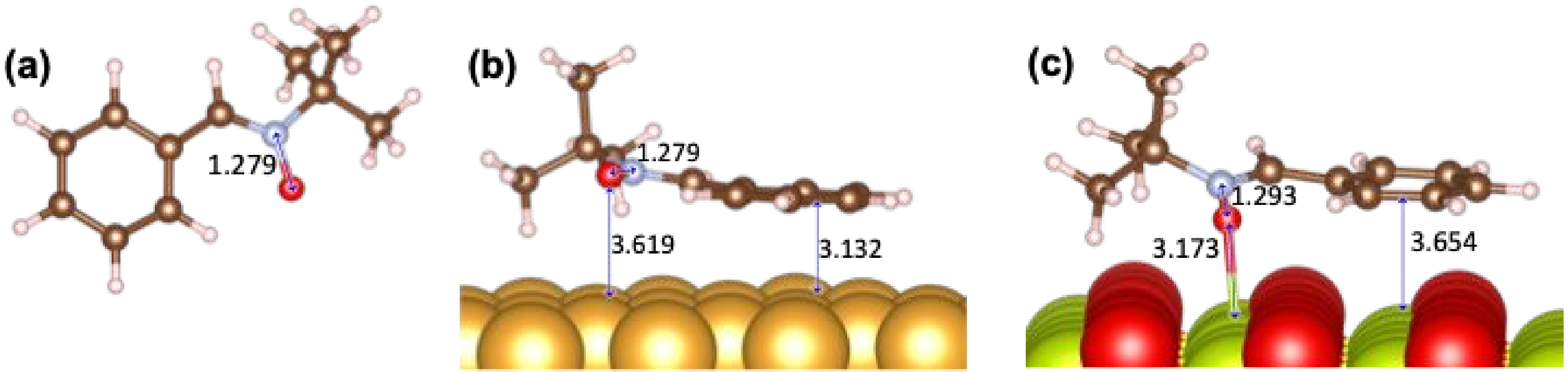

Figure S21. PBN configurations in (a) gas phase and over (b) Au(111) and (c) CeO_2(111).

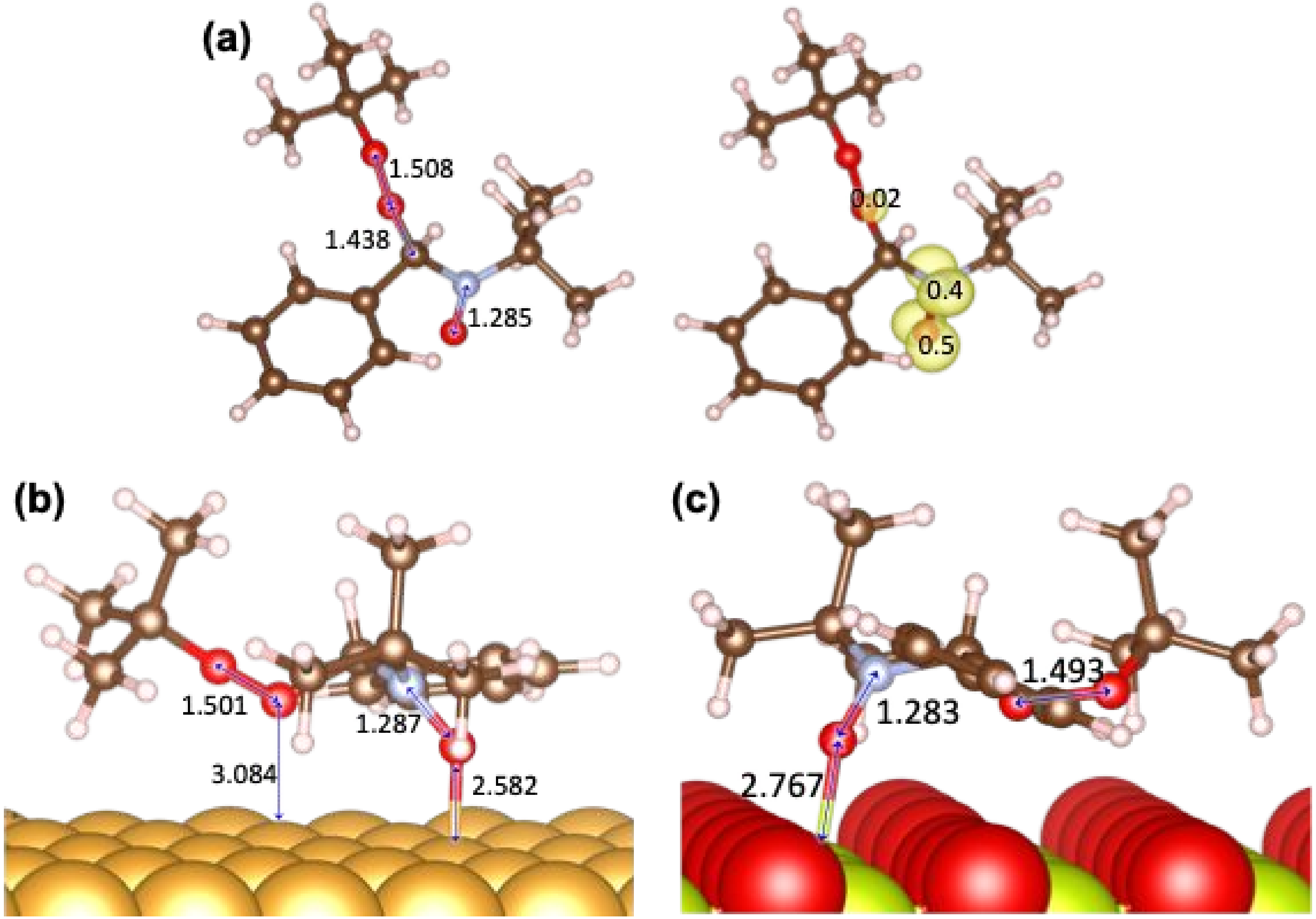

Figure S22. PBN-tBu-OO• configurations in (a) gas phase and over (b) Au(111) and (c) CeO_2(111).

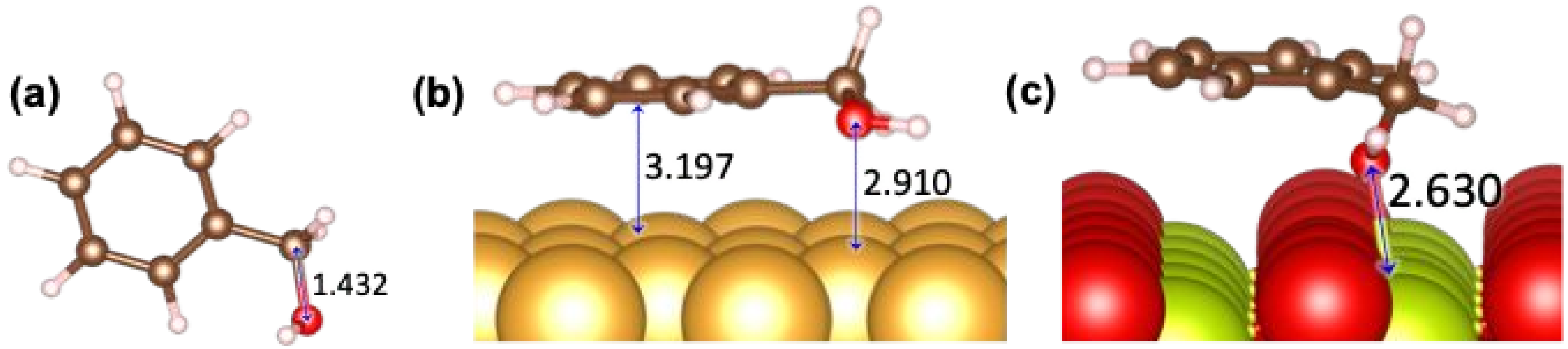

Figure S23. BnOH configurations in (a) gas phase and over (b) Au(111) and (c) CeO_2(111).

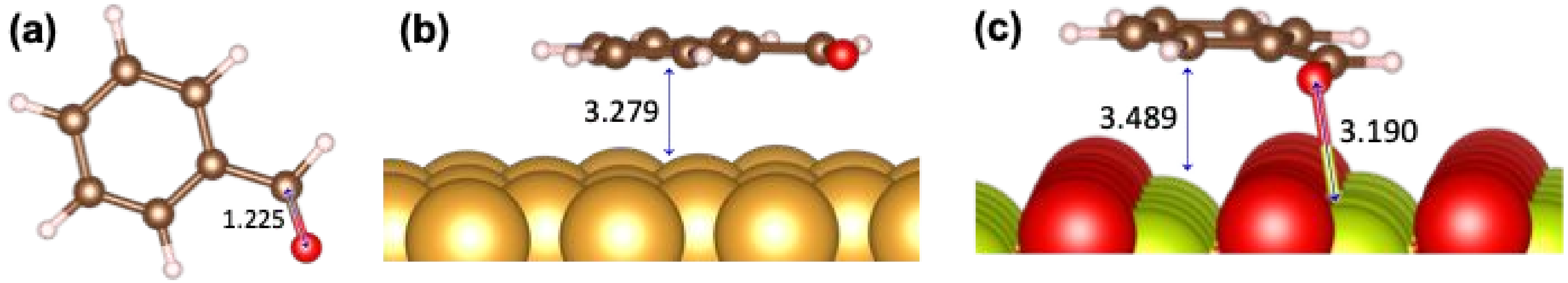

Figure S24. BnAH configurations in (a) gas phase and over (b) Au(111) and (c) CeO_2(111).

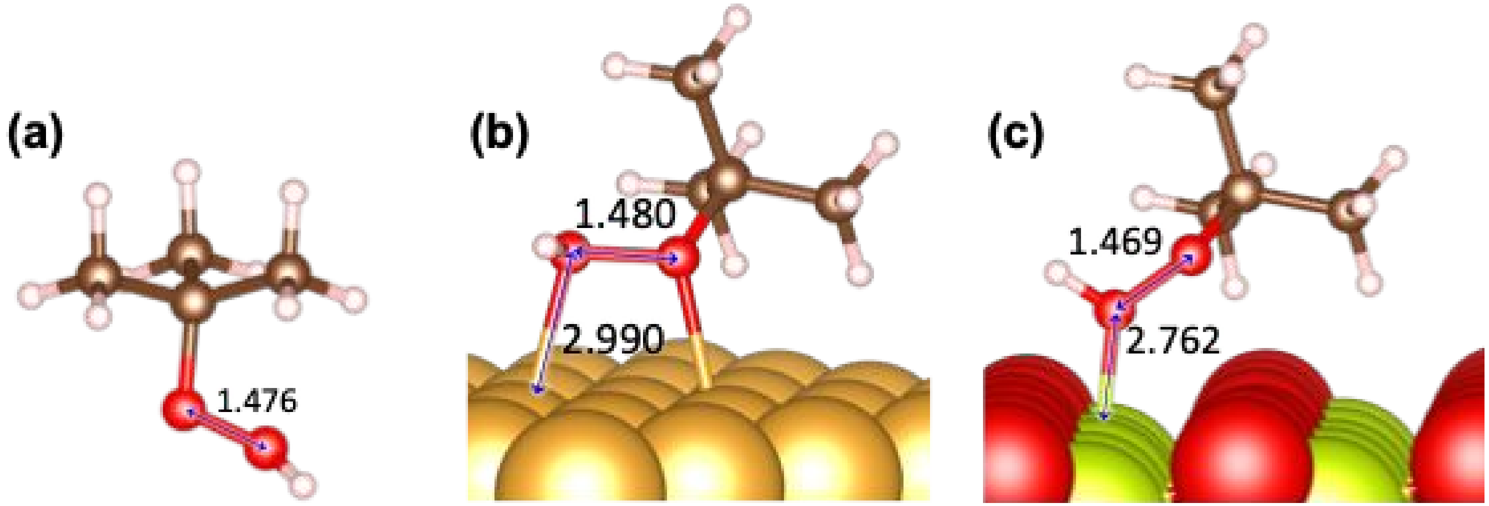

Figure S25. TBHP configurations in (a) gas phase and over (b) Au(111) and (c) CeO_2(111).

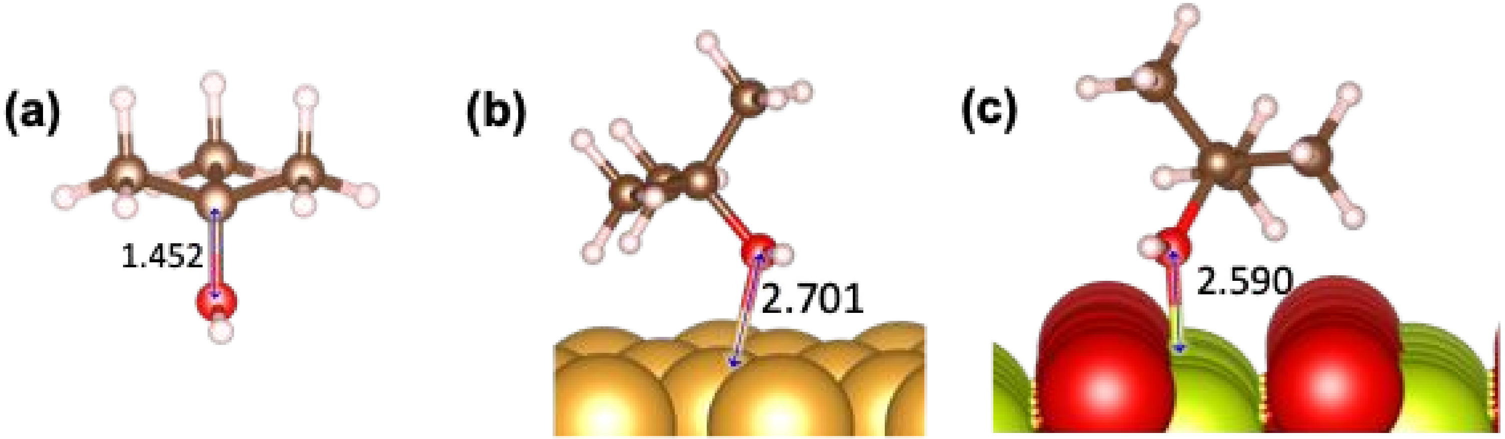

Figure S26. tBuOH onfigurations in (a) gas phase and over (b) Au(111) and (c) CeO_2(111).
